# The cost of mental disorders in Denmark: a register-based study

**DOI:** 10.1038/s44184-022-00001-y

**Published:** 2022-05-25

**Authors:** Maria Klitgaard Christensen, John J. McGrath, Natalie C. Momen, Harvey A. Whiteford, Nanna Weye, Esben Agerbo, Carsten Bøcker Pedersen, Preben Bo Mortensen, Oleguer Plana-Ripoll, Kim Moesgaard Iburg

**Affiliations:** 1 National Centre for Register-based Research, Aarhus University, Aarhus, Denmark; 2 Department of Public Health, Aarhus University, Aarhus, Denmark; 3 Queensland Brain Institute, University of Queensland, St Lucia, QLD Australia; 4 Queensland Centre for Mental Health Research, The Park Centre for Mental Health, Wacol, QLD Australia; 5 University of Queensland, School of Public Health, Herston, QLD Australia; 6 Institute of Health Metrics and Evaluation, University of Washington, Seattle, WA USA; 7 Centre for Integrated Register-based Research (CIRRAU), Aarhus University, Aarhus, Denmark; 8 The Lundbeck Foundation Initiative for Integrative Psychiatric Research (iPSYCH), Aarhus, Denmark; 9 Department of Clinical Epidemiology, Aarhus University and Aarhus University Hospital, Aarhus, Denmark

**Keywords:** Health care economics, Psychiatric disorders

## Abstract

The aim of the study was to undertake a detailed analysis of healthcare cost, public transfer payments, and income loss associated with a broad range of mental disorders in Denmark. Based on all persons living in Denmark, we identified those with a hospital diagnosis of one of 18 types of mental disorders and 10 age- and sex-matched controls per case. For each mental disorder, the outcomes were nationwide totals, cost per case, and cost per capita, investigated by sex, age strata, and the number of years after diagnosis. We found a substantial annual income loss of 5 billion Euros and excess healthcare cost of 1 billion Euros for persons with any mental disorder. Each mental disorder was associated with an income loss, excess healthcare cost, and excess public transfer payments compared to matched controls. An interactive data visualisation site with summary data is available at https://nbepi.com/cost.

## Introduction

Mental disorders account for a sizeable proportion of years lived with disability (YLDs) in high-income countries^1^. With the ageing of populations and improved interventions for infectious and nutritional disorders, the relative and absolute burden of mental disorders will increase in the decades ahead. Mental disorders often have an onset in childhood or young adulthood^2^ and besides a health loss for the individual^3^, the disorders also have a negative impact on educational attainment and participation in the labour force^4^. In addition to the impact of these disorders on the individual, there are also outcomes that can be quantified within economic frameworks. For example, cost-of-illness studies can measure and compare the economic impact of specific disorders in order to communicate the relative importance of these disorders to the public, health planners, and policy- and decision-makers^5^.

A comprehensive systematic review on the cost of mental disorders worldwide found significant costs associated with mental disorders in both high-, low- and middle-income countries^6^. Disorders such as schizophrenia and intellectual disabilities were associated with great societal cost and the ranking of mental disorder types by cost was relatively stable between different countries. However, the systematic review found a lack of data for disorders such as eating disorders, behavioural disorders, intellectual disabilities, and personality disorders. In addition, the review noted that methodological differences made between-study comparisons problematic. There is a need for studies to address these gaps to expand our understanding of the cost of mental disorders. Furthermore, cost-of-illness studies have been criticised for not providing a meaningful counterfactual alternative (e.g. what might have happened under different conditions)^7^. The use of a matched case-cohort design using national registers makes it possible to investigate the question: “What is the observed difference in cost between people with a particular disorder versus comparable people without that disorder?” Even though this measure of ‘excess’ cost does not allow causal inferences, it provides a focused examination of the association between types of mental disorders and economic outcomes.

In addition, while many studies have examined one particular type of mental disorder^4,6,8–11^, relatively few studies have presented data on a comprehensive range of mental disorders using the same methodological approach^12–14^. One of the few studies that did was a recent study by Vestergaard et al.^13^, which used individual-level register data for several cost measures to estimate the cost-of-illness for a range of brain disorders in Denmark. However, this study, which grouped mental disorders into broad categories, did not present estimates for mental disorders by sex and age, nor examine how costs change over the years following the onset of a mental disorder.

We wanted to utilise the strengths of the Danish registers and investigate the costs over a long follow-up period using a prevalence-based approach. This approach is well-suited for providing information to health planners about how costs differ in magnitude and distribution between disorders^5^. We were able to examine the costs associated with a broad range of mental disorders and to examine a range of gaps in the prior literature. We focused on healthcare cost from the healthcare provider perspective, the productivity loss for society and the individual, and public transfer payments from the government. We have previously demonstrated that mental disorders have an increased risk of subsequent somatic disorder comorbidity^15^, and thus we were interested in exploring the contribution of costs associated with (a) psychiatric services, and (b) the care of comorbid somatic disorders.

Our study was guided by several specific research questions. First, we were interested in how costs varied by mental disorder type, and how rankings of average cost per case of each type of mental disorder differed from nationwide cost for all persons of each mental disorder type in Denmark. We expected some common disorders would be less costly per case (e.g. depression) while other low prevalence disorders would be more expensive per case (e.g. schizophrenia and anorexia). Based on prior studies^13,16–18^, we expected that production loss (i.e. as measured by the difference in income) would be greater than the healthcare cost. Because the prevalence of types of mental disorders differ by sex^19^ and age-of-onset^2^, we examined how costs associated with different types of mental disorders varied by sex and age strata. Finally, previous studies investigating particular mental disorders have shown the costs vary by the time after disorder diagnosis (with the highest costs around the time of diagnosis). However, this issue has only been examined for a relatively short period^9,17,20^. Thus, we wanted to examine how costs varied over a longer (14 years) follow-up period from the first diagnosis registered in the psychiatric hospital register. Because of the comprehensive and detailed nature of our study, we also provide an interactive data visualisation site to facilitate more fine-grained analyses of our results: https://nbepi.com/cost.

## Methods

### Study population

Persons living in Denmark between 2004 and 2017 were identified using the Danish Civil Registration System^21^ and were followed from 1st of January 2004, birth or immigration (whichever occurred last) until death, 95th birthday, emigration or 23rd of April 2017 (whichever occurred first).

Cases were defined as persons with a mental disorder (referred to as index disorder hereafter) based on the definitions used in the Global Burden of Disease (GBD) study and in previous Danish register-based studies^3^. The 18 mental disorders included were: alcohol-use disorder, opioid-use disorder, cannabis-use disorder, cocaine-use disorder, amphetamine-use disorder, other drug-use disorders, schizophrenia, bipolar disorder, major depressive disorder, dysthymia, anxiety disorders, anorexia nervosa, bulimia nervosa, personality disorders, intellectual disabilities, autism spectrum disorders, ADHD and conduct disorders. A Danish modification of The International Classification of Diseases, Tenth Revision (ICD-10) was used (ICD-10 codes and the assumed earliest age of onset are available in Supplementary Table 1). Cases were identified from the first date of a diagnosis registration from either an inpatient, outpatient or emergency contact (referred to as ‘diagnosis’ hereafter) using the Danish Psychiatric Central Research Register^22^ between 1995 and 2017. The register contains information on all admissions to Danish psychiatric inpatient and outpatient facilities and emergency-room visits. Each case was randomly matched with 10 persons from the Danish general population with same sex and birthdate (+/− two months) and without the index disorder at the time of diagnosis for the case.

### Costs

A broad range of cost measures were included in the analysis, which can be divided into two perspectives: the healthcare provider perspective, and productivity loss for society (i.e. income loss). We also looked at publicly financed transfer payments (e.g. public pensions, unemployment benefits, other social financial transfers) even though these are not a cost but a redistribution of national income.

With respect to the public healthcare cost, psychiatric service cost from in- and outpatients and emergency-room contacts in hospitals were identified using data from the Diagnosis-Related Group (DRG) Psychiatric Patient Register. The DRG tariffs for Mental disorders in Denmark are average national tariffs for in- and outpatients and do not distinguish between mental disorder diagnoses and their severity. Hospital cost from somatic disorders was identified using the DRG National Patient Register^23^, which contains DRG tariffs for every somatic patient contact for specific disorders. DRG tariffs for somatic disorders are the average national operating expenses for treating patients within the same diagnostic group and are used for reimbursement of public and private hospitals as hospital treatment is free of charge for Danish citizens. The Danish Health Data Authority is responsible for estimating annual somatic and psychiatric DRGs^24^. Subsidised prescription costs were identified using the National Prescription Registry^25^ and were calculated by subtracting the out-of-pocket patient cost from the nationwide service cost. Healthcare cost from primary healthcare providers (general practitioners, practising medical specialists, psychologists, dentists, physiotherapists, chiropodists and chiropractors) with an agreement with the tax-funded healthcare system was identified using the Danish National Health Service Register^26^. The combined healthcare cost was calculated as the annual sum of hospital costs from mental disorders and somatic disorders, subsidised prescription cost, and primary healthcare service cost for every individual included in the study.

With respect to productivity loss, this was measured as the difference in personal income excluding public transfer payments for cases and controls (called income loss hereafter). The personal income before tax was identified from the Income Statistics Register^27^. Publicly financed transfer payments were also identified from the Income Statistics Register. The transfer payments consist of unemployment benefits, social assistance, state educational grants, housing benefits, child and youth benefits, public old-age pension, disability pension, flexi-job (wage subsidy for long-term partially disabled persons), and early retirement. These are publicly financed and do not represent any real resource consumption in society, but are transfers of resources.

The patient out-of-pocket prescription cost was identified from the National Prescription Registry. Because out-of-pocket cost is small in Denmark, we include these results in Supplementary Table 3.

### Statistical analysis

For each specific mental disorder, the economic outcomes were nationwide annual cost (i.e. all cases in the registers), average annual cost per case and annual cost per capita estimated as an average over the 14 year period. The healthcare costs were expressed both in annual cost for cases for each type of mental disorder (absolute cost) and the annual difference in costs for cases compared to controls (excess cost). The income loss and public transfer payments were expressed as the difference between cases and controls. Persons diagnosed with more than one type of mental disorder contributed as a case for each relevant index disorder. For each mental disorder, we also calculated costs for each sex, age strata and years after first diagnosis. For the cost analyses by years after diagnosis, the cases were identified between 2004 and 2017 instead of 1995 to 2017 due to a lack of data availability of the cost measures prior to the year 2004. The per capita calculations were computed by dividing the nationwide annual cost for each mental disorder by the Danish population in 2017 from Statistics Denmark according to sex and age strata.

All cost estimates were adjusted for inflation in Denmark from the year the cost occurred to 2017 using Gross Domestic Product deflators from the World Bank and were converted to Euro using the average exchange rate for 2017 from the European Central Bank.

Data analyses were performed using R version 4.0.4 on the secure platform of Statistics Denmark. A data visualisation site with summary data is available for download at the following link: https://nbepi.com/cost.

### Ethics approval

The Danish Data Protection Agency, the Danish Health Data Authority, and Statistics Denmark approved of this study. Informed consent is not required for register-based studies according to Danish law.

## Results

Between 1995 and 2017, a total of 447,209 persons (238,659 females, 208,550 males) were diagnosed with at least one mental disorder. The median age at diagnosis of any mental disorder was higher for females compared with males (32 and 30 years, respectively). The number of cases and the median age at diagnosis for each type of mental disorder are shown in Table 1, and the number of cases and the median age for the period 2004 to 2017 can be found in Supplementary Table 2.

**Table 1:** Description of the study population.

	Females		Males		Total	
	Cases	Median age	Cases	Median age	Cases	Median age
Any mental disorder	238,659	32	208,550	30	447,209	31
Alcohol-use disorder	18,760	47	36,210	44	54,970	45
Opioid-use disorder	1958	39	3847	36	5805	37
Cannabis-use disorder	4284	20	13,230	23	17,514	23
Cocaine-use disorder	520	23	1556	26	2076	26
Amphetamine-use disorder	726	22	1915	26	2641	25
Other drug-use disorders	4035	49	3430	38	7465	45
Schizophrenia	16,642	37	23,043	34	39,685	35
Bipolar disorder	17,381	43	11,964	43	29,345	43
Major depressive disorder	121,403	38	71,405	39	192,808	38
Dysthymia	4511	39	2551	38	7062	39
Anxiety disorders	75,603	29	48,935	29	124,538	29
Anorexia nervosa	10,082	15	629	11	10,711	15
Bulimia nervosa	5877	21	135	22	6012	21
Personality disorders	56,691	28	30,296	32	86,987	29
Intellectual disabilities	9423	19	14,222	13	23,645	15
Autism spectrum disorders	9175	6	26,053	5	35,228	5
ADHD	17,075	10	36,320	7	53,395	8
Conduct disorders	546	11	1753	11	2299	11

The number of cases and the median age of mental disorder diagnosis in years by mental disorder and sex between 1995 and 2017.

### Costs associated with types of mental disorders

**Fig. 1:**
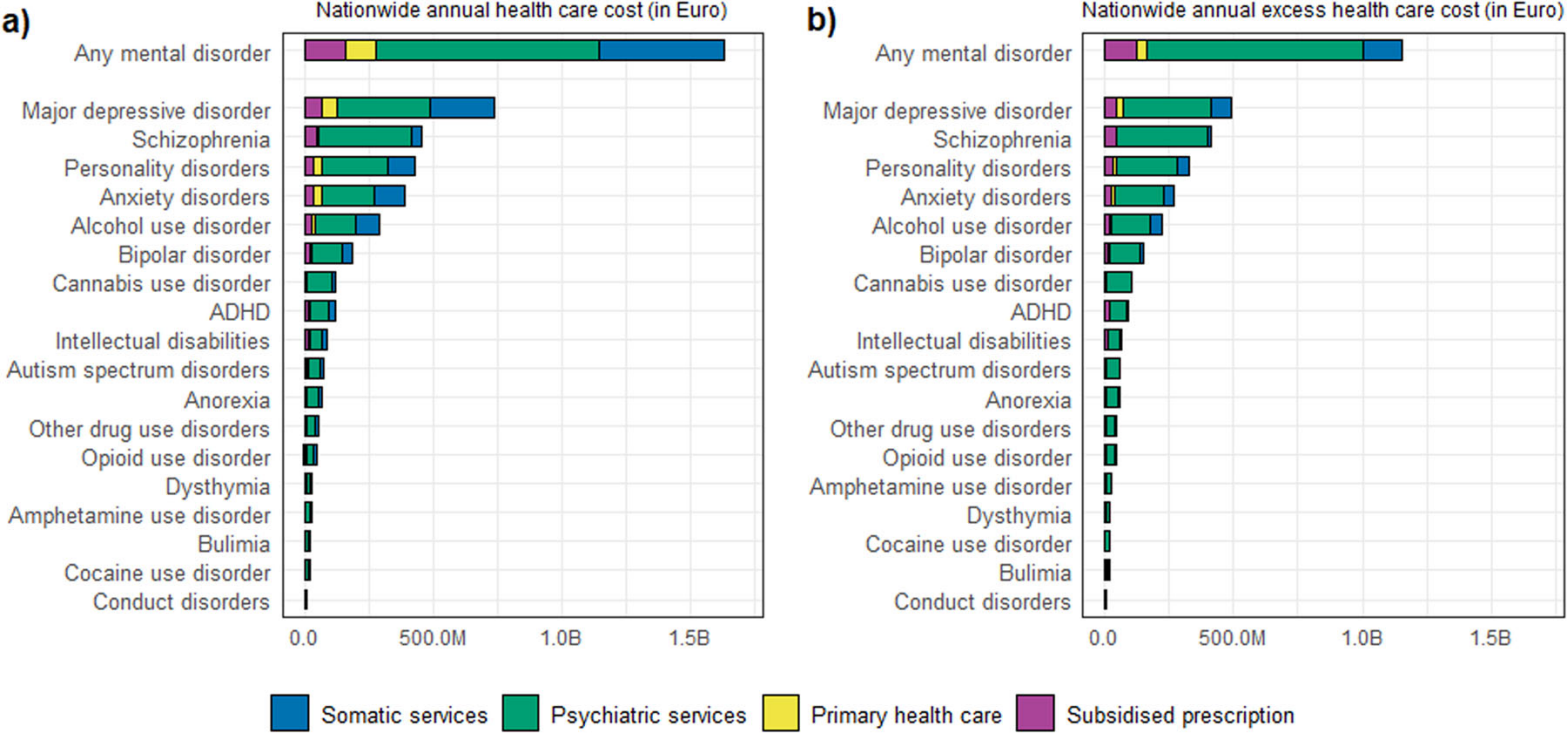
Ranking of mental disorders according to nationwide healthcare cost. Any mental disorder and 18 types of mental disorders ranked according to (**a**) nationwide annual health care cost, and (**b**) nationwide annual excess healthcare cost (Euro 2017). The contribution of different cost categories are indicated by the colours.

Mental disorders were associated with substantial nationwide annual healthcare cost over the study period. Overall, those with any mental disorder had a nationwide annual healthcare cost for 1.63 billion (B) Euro. This corresponded to 284 Euro per capita per year for any mental disorder. Figure 1a shows the ranking of each of the 18 mental disorders, from highest to lowest nationwide annual cost and the share of the different healthcare cost categories. Major depressive disorder (736 million [M] Euro), schizophrenia (457 M Euro), and personality disorders (430 M Euro) ranked the highest in nationwide annual healthcare cost, while conduct (7 M Euro) and cocaine-use disorder (18 M Euro) had the lowest annual cost. Psychiatric services were the predominant healthcare cost, but cost from somatic services was also substantial for the disorders at the top of the range, while costs from subsidised prescriptions and primary healthcare were notable.

With respect to the nationwide annual excess healthcare cost (Fig. 1b), which is the difference in cost between cases and controls, persons with mental disorders had considerably higher cost for each disorder type than the matched controls, as expected. There were minor changes between the lower ranked mental disorders going from absolute to excess cost. Compared to absolute cost, excess cost for psychiatric services did not change notably, but the three other cost categories (somatic services, primary health care and subsidised prescriptions) were markedly smaller. Of interest, excess cost for mental disorders in the top half of the rankings (e.g. major depressive disorder, personality disorders, alcohol-use disorder) were associated with considerable cost related to somatic services.

**Fig. 2:**
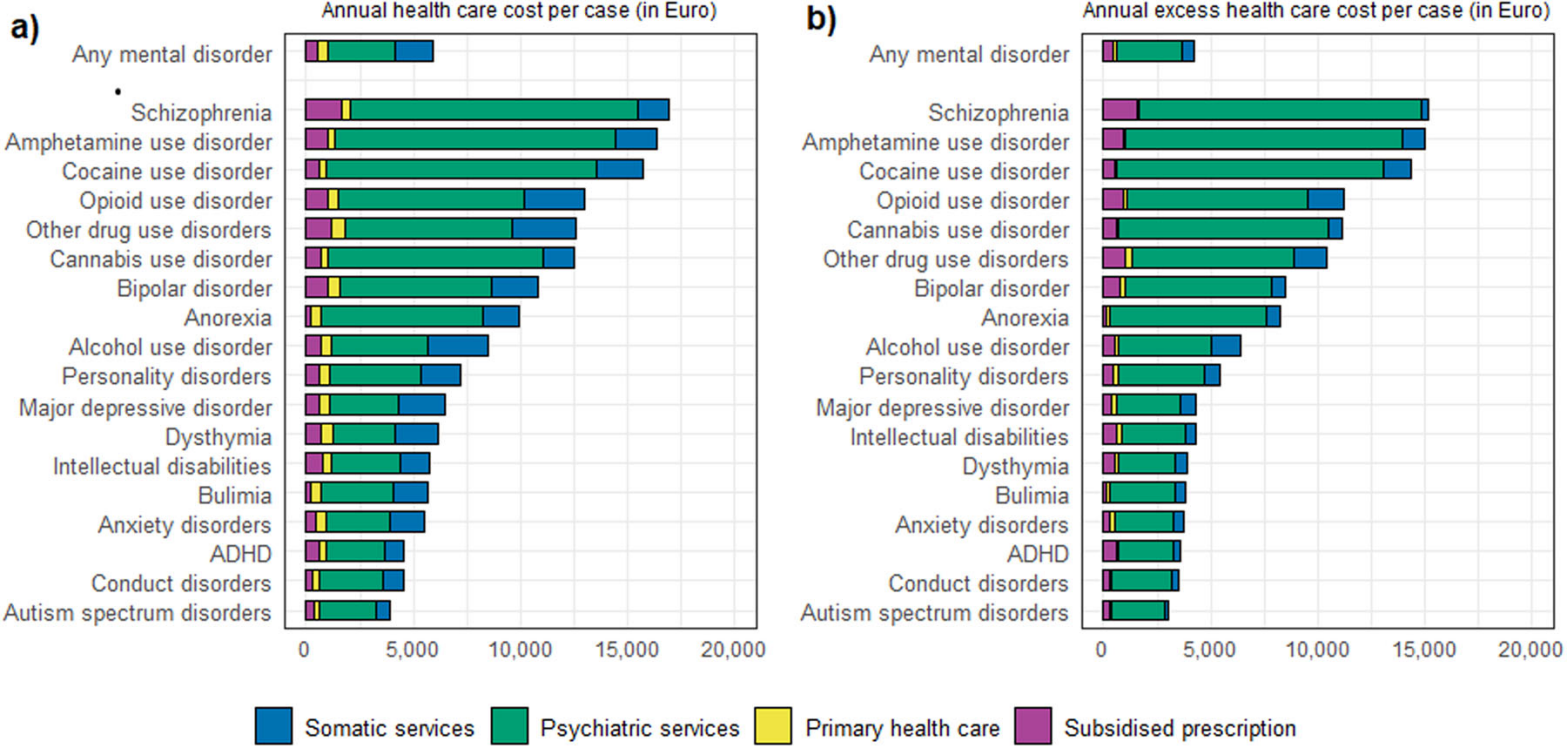
Ranking of mental disorders according to healthcare cost per case. Any mental disorder and 18 types of mental disorders ranked according to (**a**) annual healthcare cost per case, and (**b**) annual excess healthcare cost per case (Euro 2017). The contribution of the different cost categories is indicated by the colours.

In general, the more prevalent mental disorders were dominant in the ranking of nationwide annual and nationwide annual excess healthcare cost, however, the ranking of the nationwide cost also reflected that some disorders had a relatively high annual cost per case (i.e. schizophrenia ranking in the top [16,910 Euro per case]). Figure 2 shows the absolute and excess healthcare cost per case, with schizophrenia and drug-use disorders ranking in the top.

**Fig. 3:**
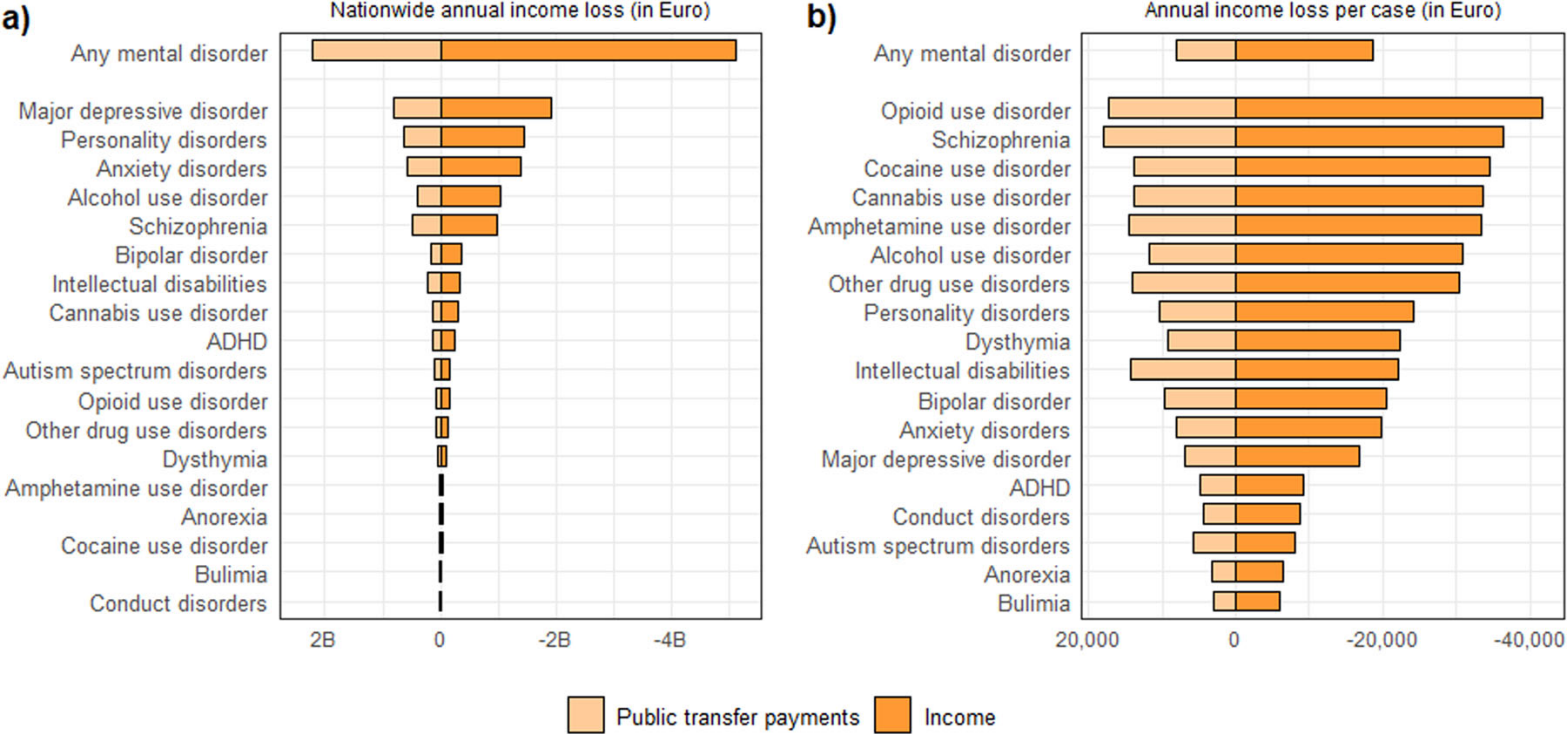
Ranking of mental disorders according to nationwide income loss and income loss per case. Any mental disorder and 18 types of mental disorders ranked according to (**a**) nationwide annual income loss, and (**b**) nationwide annual excess public transfer payments (Euro 2017). The income loss and excess public transfer payments are indicated by the colours.

A similar pattern was found for nationwide annual income loss (Fig. 3a). The negative income values for the nationwide annual income loss correspond to a lower annual income for cases compared to controls. Each of the 18 mental disorders was associated with an annual income loss. The combined annual income loss for any mental disorder was 5.12 billion (B) Euro.

Major depressive disorder, personality disorders, and anxiety disorders accounted for the highest amount (−1.91 B Euro, −1.45 B Euro, −1.39 B Euro, respectively), while conduct disorders, bulimia and cocaine-use disorder accounted for the lowest nationwide annual income loss (−14 M Euro, −25 M Euro, −39 M Euro, respectively). This corresponded to an annual income loss of −891 Euro per capita in Denmark for any mental disorder.

The nationwide annual amount of public transfer payments is also displayed in Fig. 3a. Each of the 18 mental disorders received excess public transfer payments compared to the controls. Drug-use disorders and schizophrenia had the highest annual income loss per case (ranging from −30,377 to −41,721 Euro), while the loss per case for any mental disorder was −18,658 Euro (Fig. 3b). Additional details of the nationwide annual income loss, mean annual income loss per case and per capita by disorder type and sex, and the potential income loss in percentages by mental disorder type can be found in the Supplementary Figs. 8–11 and Supplementary Table 4.

### Costs by sex

Sex differences were found in the cost ranking for the 18 mental disorder types. Major depressive disorder (484 M Euro) and schizophrenia (260 M Euro) accounted for the highest nationwide annual absolute healthcare cost for females and males, respectively. Even though psychiatric services cost featured prominently, somatic services contributed a large proportion of the nationwide cost for females with major depressive disorder (160 M Euro). In addition, alcohol-use disorder ranked higher for males than females, while anorexia and bulimia ranked higher for females than males. The pattern persisted when we examined the nationwide annual excess healthcare cost. For the healthcare cost per case, the sex-specific rankings were similar in the top half with schizophrenia and drug-use disorders ranking highest for both. See Supplementary Figs. 1–5. Major depressive disorder had the highest nationwide annual income loss, followed by personality disorders and anxiety disorder for females (−977 M, −755 M and −700 M Euro, respectively), while major depressive disorder, alcohol-use disorder and personality disorders accounted for the highest nationwide annual income loss for males (−935 M, −791 M, −693 M Euro, respectively). Persons with opioid-use disorder or schizophrenia had on average the highest income loss per case for both sexes. The nationwide annual income loss and the annual income loss per case and per capita by sex are displayed in Supplementary Figs. 10–12.

### Costs by age

**Fig. 4:**
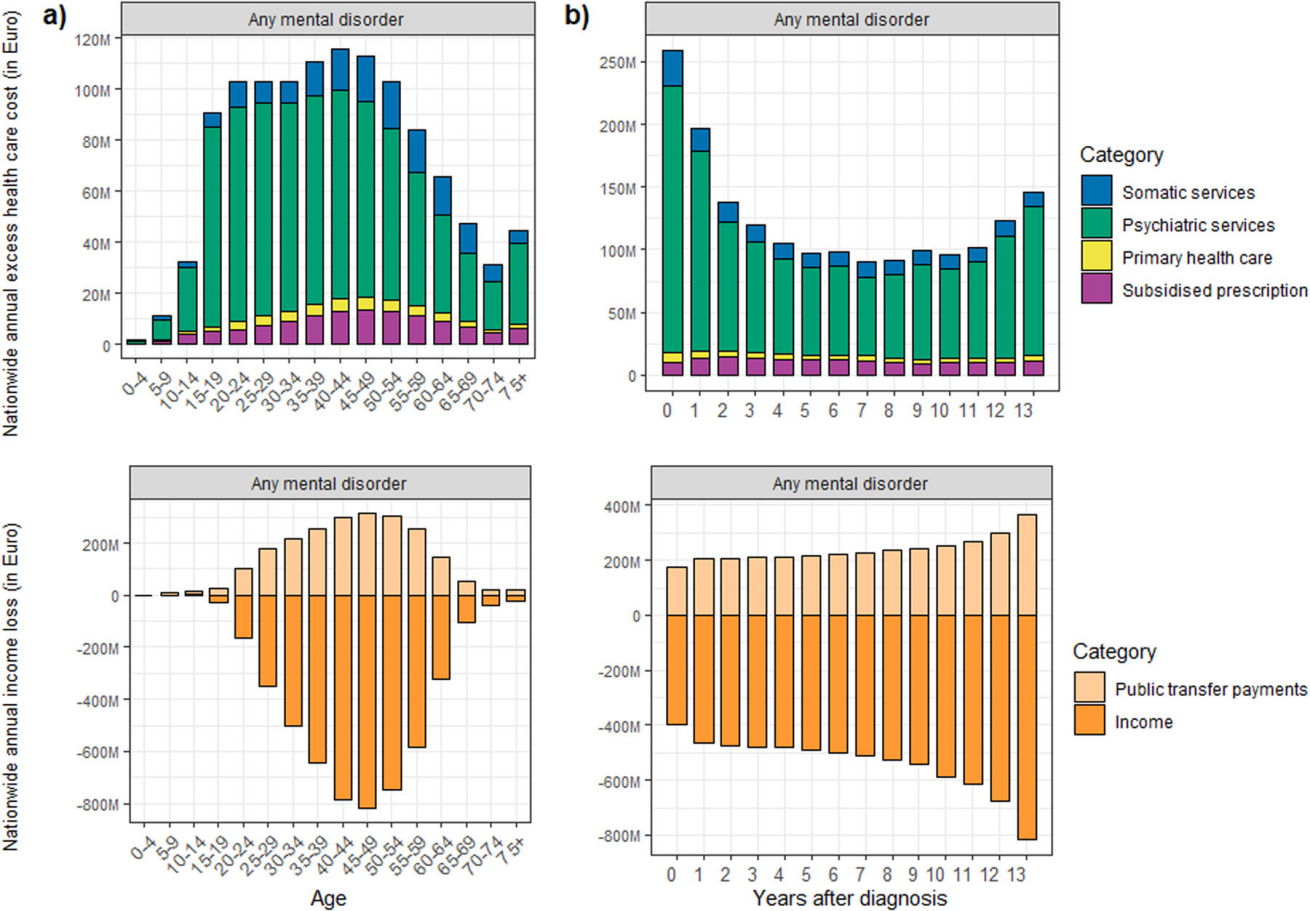
Healthcare cost and income loss by age and time since diagnosis. Nationwide annual excess healthcare cost and nationwide annual income loss (Euro 2017) for any mental disorder by (**a**) age group, and (**b**) years after diagnosis.

Persons with any mental disorder had higher nationwide annual excess healthcare cost compared with the controls for each age group (Fig. 4a). The cost was highest and on a similar level from age 15 to 49 years. Thus, the annual excess cost was already high for adolescents. The pattern of findings was comparable for the nationwide annual cost (Supplementary Fig. 13). The annual absolute healthcare cost per case increased steadily from age 5 to 74. Notably, the cost contribution from somatic services increased with age, while the cost from psychiatric services fell (Supplementary Fig. 14). The nationwide and per case annual absolute and excess healthcare cost by mental disorder type and age can be found in Supplementary Figs. 16–22. We found an annual income loss per case in all age groups, and the highest loss was found in middle age (40–54 years). Thus, the difference in income between cases and controls increased until middle age, after which it declined again. On a population-level, this corresponded to a substantial nationwide annual income loss for cases with any mental disorder.

### Costs by time since diagnosis

With respect to the costs in the years after diagnosis, we found a general pattern with the highest healthcare cost in the first year after a hospital diagnosis with falling cost over the follow-up period (Fig. 4b and Supplementary Figs. 12–14). Notably, the excess healthcare cost persisted even 13 years after the time of diagnosis, and the nationwide annual income loss also increased over time (Fig. 4b). The increasing income loss reflected that cases and controls got older and that the controls experienced an appreciable higher increase in income than cases. In contrast, cases received increasing excess public transfer payments as years passed following diagnosis. The disorder-specific figures can be found in the supplement (Supplementary Figs. 21–26).

## Discussion

In this register-based study, we have, to the best of our knowledge, provided the most comprehensive and detailed description of the costs associated with mental disorders. We found a substantial nationwide annual income loss of 5 billion Euros and nationwide excess healthcare cost of 1 billion Euros for persons with any mental disorder. In the discussion, we will focus on four key findings.

First, we found variation in the costs among the different types of mental disorders, and variation in the rankings of mental disorders according to nationwide cost versus per case cost. There were discrepancies between the ranking we found in cost per case and the ranking found in a systematic review of the cost of mental disorders (e.g. developmental disorders and intellectual disabilities ranged in the bottom half in our study while in the top in the systematic review^6^). However, the systematic review was based on heterogeneous articles from different countries and with different levels of cost details which could explain the different results. In addition, another register-based study estimating the costs in cases with prevalent brain disorders in Denmark also found depression to be associated with the highest nationwide cost^13^, but their use of broad disorder categories does not allow a direct comparison.

Second, in keeping with previous studies^10,13,16–18^, we found higher cost from productivity or income loss than from healthcare cost. Nevertheless, the nationwide annual healthcare cost among persons with mental disorders were still substantial. Both in terms of nationwide absolute cost, but also in terms of nationwide excess cost, where we estimated the difference between cases and their matched controls and found the prominent excess cost for each of the 18 mental disorder types. The estimates based on excess cost lend weight to the hypothesis that the healthcare costs are directly and/or indirectly associated with the index mental disorder.

Third, there were differences in the ranking by sex and by age. Major depressive disorder and schizophrenia accounted for the highest healthcare cost for females and males, respectively. Notably, the costs of mental disorders were prominent in young people as the nationwide annual excess health care cost was near the top-level already in teenagers (around age 15 to 19 years).

Fourth, the excess healthcare cost was numerically higher for cases even 13 years after the first registration of a psychiatric hospital diagnosis. Other studies have also found excess cost among cases in the years after a mental disorder diagnosis^9,13,17,20^, but none had, to the best of our knowledge, the same lengthy follow-up time. Future studies could investigate if the decline over time could be due to recovery for some cases and also, how the costs vary between those who recover versus those with persistent psychiatric care contacts.

This study has several important strengths. The analyses were based on high-quality data from national registers with prospectively collected information on all citizens in Denmark. Healthcare services within hospitals are free of charge in Denmark entailing minimal selection bias in regard to treatment access. We estimated costs encompassed by an individual regardless of the underlying cost drivers and did so for a wider range of mental disorders than previous studies, creating a comprehensive overview of the cost of mental disorders in a Danish setting. We applied a prevalence-based approach to be able to identify different cost components and their nationwide cost in society and to inform health planners, and policy- and decision-makers about the economic impact of specific mental disorders.

The study also has several limitations. First, only persons seeking help within psychiatric hospitals were included as cases in this study. Prior studies have suggested that mild cases of disorders such as depression are treated only in general practice^28^, or not treated at all. This bias would inflate the costs for the controls, and this could result in an underestimation of the excess costs associated with mental disorders. We could not include all potentially relevant costs in our analyses because of lack of data availability across the period of observation. Costs from other sectors such as social services, criminal justice, leisure and transportation time, and informal care from family and caregivers could have shed light on the costs from additional perspectives. For example, education service cost have been shown to be prominent in childhood disorders^29^. As with many modern registers, we have limited follow-up time in older ages for mental disorders mostly diagnosed in childhood. This could affect the validity of the cost according to age for these mental disorder types since persons diagnosed later in life might be dissimilar to the average case. With respect to generalisability, our cost estimates may not reflect other countries with different healthcare systems and labour market structures and rights of employees. Finally, as this is a descriptive study, it is not possible to conclude that the excess costs are caused by the index mental disorder—unmeasured confounding (e.g. socioeconomic factors) may influence the association between mental disorders and economic outcomes. Future studies should seek to investigate the mechanisms behind the observed associations and, if these are modifiable, develop cost-effective interventions to tackle underlying cost drivers.

Our findings can be used to inform health planners and policy- and decision-makers of the economic impact of a wide range of mental disorder types in Denmark. We believe our results emphasise the need to design interventions and better treatment options for persons with mental disorders and to help them participate in the labour force and thereby reducing the income loss for the individual and for society.

### Supplementary information

Supplementary Material

## Data Availability

The individual-level data used for this study are not publicly available but can be obtained by application to The Danish Health Data Authority (www.sundhedsdatastyrelsen.dk) and Statistics Denmark (https://www.dst.dk/en).

## Code availability

Data management and data analysis were performed on the secure platform of Statistics Denmark, and the code is not publicly available.
