# Supplementary material for: The cost of mental disorders in Denmark: a register-based study
Source: Npj Ment Health Res. 2022 May 25;1:1. doi: 10.1038/s44184-022-00001-y (PMC10938857; doi:10.1038/s44184-022-00001-y)
Supplement: Supplementary file 1 — Supplementary Material [file 44184_2022_1_MOESM1_ESM.pdf]

Supplementary material to ‘The Cost of Mental Disorders in Denmark: a register-based study’

Contents

Supplementary Tables 2
Supplementary Figures 7
Ranking figure per capita 7
Figures by age and years after diagnosis for any mental disorder 18
Figures by age and mental disorder type 21
Figures by years after diagnosis and mental disorder type 28

Supplementary Tables

Supplementary Table 1: The table show the ICD-10 codes for the included mental disorders and the assumed earliest possible age at onset in years.

Disorders	ICD-10 codes	Earliest age of onset (years)
Any mental disorder	F10.2, F11.2, F12.2, F13.2, F14.2, F15.2, F16.2, F18.2, F20, F30-F33 (except F31.7), F34.0, F34.1, F40-F42, F43.1, F50.0- F50.2, F60, F70-F79, F84, F90, F91.0- F91.2, F91.8, F93.0- F93.2, F93.8, Z81.0	1
Alcohol use disorder	F10.2	10
Opioid use disorder	F11.2	10
Cannabis use disorder	F12.2	10
Cocaine use disorder	F14.2	10
Amphetamine use disorder	F15.2	10
Other drug use disorders	F13.2, F16.2, F18.2	10
Schizophrenia	F20	10
Bipolar disorder	F30, F31 (except F31.7), F34.0	10
Major depressive disorder	F32, F33	10
Dysthymia	F34.1	10
Anxiety disorders	F40, F41, F42, F43.1, F93.0-F93.2, F93.8	1
Anorexia nervosa	F50.0, F50.1	1
Bulimia nervosa	F50.2	1
Personality disorders	F60	10
Intellectual disabilities	F70-F79, Z81.0	1
Autism spectrum disorders	F84	1
ADHD	F90	1
Conduct disorders	F91.0-F91.2, F91.8	1

Supplementary Table 2. Number of cases and median age at hospital diagnosis in years by mental disorder and sex between 2004 and 2017.

	Females		Males		Total	
	Cases no.	Median age	Cases Co.	Median age	Cases no.	Median age
All cases	191,678	29	172,319	26	363,997	28
Alcohol use disorder	13,840	45	26,774	42	40,614	43
Opioid use disorder	1,350	37	2,759	34	4,109	35
Cannabis use disorder	3,779	17	11,465	21	15,244	20
Cocaine use disorder	457	22	1,386	26	1,843	25
Amphetamine use disorder	596	21	1,650	24	2,246	23
Other drug use disorders	2,833	47	2,604	36	5,437	42
Schizophrenia	14,010	34	19,764	32	33,774	33
Bipolar disorder	14,165	40	9,831	40	23,996	40
Major depressive disorder	94,907	34	56,053	37	150,960	35
Dysthymia	2,890	33	1,720	33	4,610	33
Anxiety disorders	61,236	26	40,502	26	101,738	26
Anorexia nervosa	7,860	12	512	10	8,372	12
Bulimia nervosa	4,023	17	99	17	4,122	17
Personality disorders	39,996	23	19,143	27	59,139	24
Intellectual disabilities	7,871	16	11,902	11	19,773	13
Autism spectrum disorders	8,558	5	23,245	4	31,803	5
ADHD	16,824	10	34,717	7	51,541	8
Conduct disorders	374	8	1,161	8	1,535	8

Supplementary Table 2a: Mean annual cost for cases and controls for 18 mental disorder types between 2004 and 2017. All estimates are expressed in Euro for the year 2017.

	Cases						Controls					
	Health care services mean (s.d.)		Income mean (s.d.)		Public transfer payments mean (s.d.)		Health care services mean (s.d.)		Income mean (s.d.)		Public transfer payments mean (s.d.)	
All cases	5,948	(27,874)	14,153	(31,776)	14,758	(12,063)	1,733	(2,314)	32,811	(34,893)	6,667	(5,388)
Alcohol use disorder	8,448	(37,361)	13,256	(37,202)	19,126	(11,181)	2,063	(3,171)	44,024	(46,727)	7,259	(5,159)
Opioid use disorder	13,004	(36,549)	4,680	(15,438)	23,272	(9,572)	1,787	(8,420)	46,401	(24,669)	6,027	(4,360)
Cannabis use disorder	12,484	(79,404)	6,560	(17,041)	19,060	(11,123)	1,371	(2,012)	40,142	(31,597)	5,129	(3,448)
Cocaine use disorder	15,669	(68,870)	8,955	(41,158)	19,074	(11,046)	1,363	(1,647)	43,519	(20,944)	5,165	(3,354)
Amphetamine use disorder	16,382	(68,150)	8,139	(49,263)	20,092	(11,001)	1,419	(2,118)	41,647	(24,348)	5,466	(3,542)
Other drug use disorders	12,548	(65,112)	7,553	(19,167)	22,441	(9,754)	2,111	(2,229)	37,929	(24,731)	8,287	(5,652)
Schizophrenia	16,910	(69,563)	4,042	(20,152)	24,662	(9,654)	1,777	(2,510)	40,425	(42,523)	6,735	(4,832)
Bipolar disorder	10,835	(34,752)	14,533	(33,302)	18,723	(10,987)	2,325	(2,648)	35,111	(82,549)	9,075	(5,666)
Major depressive disorder	6,453	(20,568)	17,284	(34,938)	15,482	(11,265)	2,142	(2,638)	34,035	(57,820)	8,483	(5,611)
Dysthymia	6,133	(16,146)	13,537	(23,926)	17,703	(11,070)	2,213	(3,766)	35,906	(33,261)	8,365	(5,368)
Anxiety disorders	5,541	(17,891)	14,801	(28,216)	14,835	(11,972)	1,790	(4,441)	34,519	(47,594)	6,684	(4,780)
Anorexia nervosa	9,919	(41,519)	16,289	(30,953)	9,701	(10,740)	1,701	(2,635)	22,759	(18,694)	6,340	(4,130)
Bulimia nervosa	5,680	(17,863)	24,567	(42,105)	10,637	(10,611)	1,877	(2,225)	30,634	(36,130)	7,710	(3,449)
Personality disorders	7,210	(35,324)	13,096	(23,551)	17,482	(11,662)	1,802	(2,343)	37,328	(27,921)	7,004	(4,417)
Intellectual disabilities	5,724	(41,103)	2,069	(7,141)	18,963	(14,916)	1,427	(2,085)	24,074	(26,426)	4,726	(4,868)
Autism spectrum disorders	3,942	(37,832)	3,091	(17,448)	7,895	(11,932)	906	(1,926)	11,187	(18,447)	2,151	(3,151)
ADHD	4,551	(29,457)	6,069	(18,076)	7,709	(10,628)	986	(2,377)	15,348	(23,366)	2,845	(3,481)
Conduct disorders	4,549	(21,995)	8,138	(14,885)	7,828	(10,281)	1,011	(2,046)	16,835	(16,665)	3,428	(3,459)

Supplementary Table 3: Mean annual excess cost per case for specific health care cost and by mental disorder type between 2004 and 2017 (i.e. difference in persons diagnosed with a mental disorder compared with persons without the disorder of interest). All estimates are expressed in Euro for the year 2017.

	Somatic services mean (s.d.)		Psychiatric services mean (s.d.)		Primary health care* mean (s.d.)		Subsidised prescription mean (s.d.)		Out-of-pocket prescription mean (s.d.)	
All cases	566	(6,328)	3,049	(26,944)	163	(586)	436	(1,622)	114	(293)
Alcohol use disorder	1,328	(7,725)	4,378	(36,420)	161	(590)	519	(1,690)	137	(357)
Opioid use disorder	1,714	(7,560)	8,424	(36,333)	176	(623)	903	(2,865)	479	(952)
Cannabis use disorder	591	(5,301)	9,823	(79,119)	87	(422)	612	(1,911)	143	(421)
Cocaine use disorder	1,269	(6,665)	12,395	(68,385)	107	(442)	535	(1,815)	233	(608)
Amphetamine use disorder	1,064	(6,202)	12,860	(67,660)	146	(546)	893	(2,312)	241	(555)
Other drug use disorders	1,551	(7,846)	7,586	(64,452)	309	(742)	991	(2,391)	360	(596)
Schizophrenia	286	(5,967)	13,198	(69,122)	113	(554)	1,538	(2,953)	183	(382)
Bipolar disorder	642	(7,271)	6,856	(33,733)	211	(646)	801	(1,892)	185	(319)
Major depressive disorder	698	(7,247)	3,022	(19,019)	192	(622)	400	(1,465)	126	(290)
Dysthymia	565	(6,132)	2,640	(14,920)	223	(645)	491	(1,517)	141	(311)
Anxiety disorders	507	(5,676)	2,713	(17,087)	204	(594)	326	(1,596)	106	(275)
Anorexia nervosa	631	(5,397)	7,299	(40,804)	134	(560)	153	(1,034)	48	(205)
Bulimia nervosa	444	(4,834)	3,068	(16,874)	144	(528)	147	(967)	56	(218)
Personality disorders	669	(5,785)	4,064	(34,610)	197	(601)	478	(1,673)	124	(323)
Intellectual disabilities	462	(5,263)	2,965	(40,529)	226	(805)	644	(1,991)	145	(332)
Autism spectrum disorders	156	(3,707)	2,504	(37,423)	92	(517)	284	(1,441)	78	(237)
ADHD	325	(3,984)	2,564	(29,106)	106	(434)	570	(1,460)	165	(286)
Conduct disorders	383	(3,583)	2,808	(21,467)	82	(379)	265	(1,226)	78	(251)

* General practitioners, practising medical specialists, psychologists, dentists, physiotherapists, chiropodists and chiropractors.

Supplementary Table 4: The difference in income between cases and controls as a percentage of the potential income (i.e. measured as the average income for the controls) by mental disorder type between 2004 and 2017.

	Income loss, %
All cases	68.8 %
Alcohol use disorder	76.1 %
Opioid use disorder	91.3 %
Cannabis use disorder	84.9 %
Cocaine use disorder	83.7 %
Amphetamine use disorder	84.0 %
Other drug use disorders	83.4 %
Schizophrenia	90.1 %
Bipolar disorder	71.3 %
Major depressive disorder	64.9 %
Dysthymia	71.8 %
Anxiety disorders	68.6 %
Anorexia nervosa	56.6 %
Bulimia nervosa	50.8 %
Personality disorders	73.0 %
Intellectual disabilities	86.7 %
Autism spectrum disorders	77.8 %
ADHD	70.0 %
Conduct disorders	66.7 %

Supplementary Figures

Ranking figure per capita

Supplementary Figure 1: Ranked annual absolute and excess health care cost per capita (Euro 2017) for 18 mental disorder types. The share of the different cost categories are indicated by the colours.

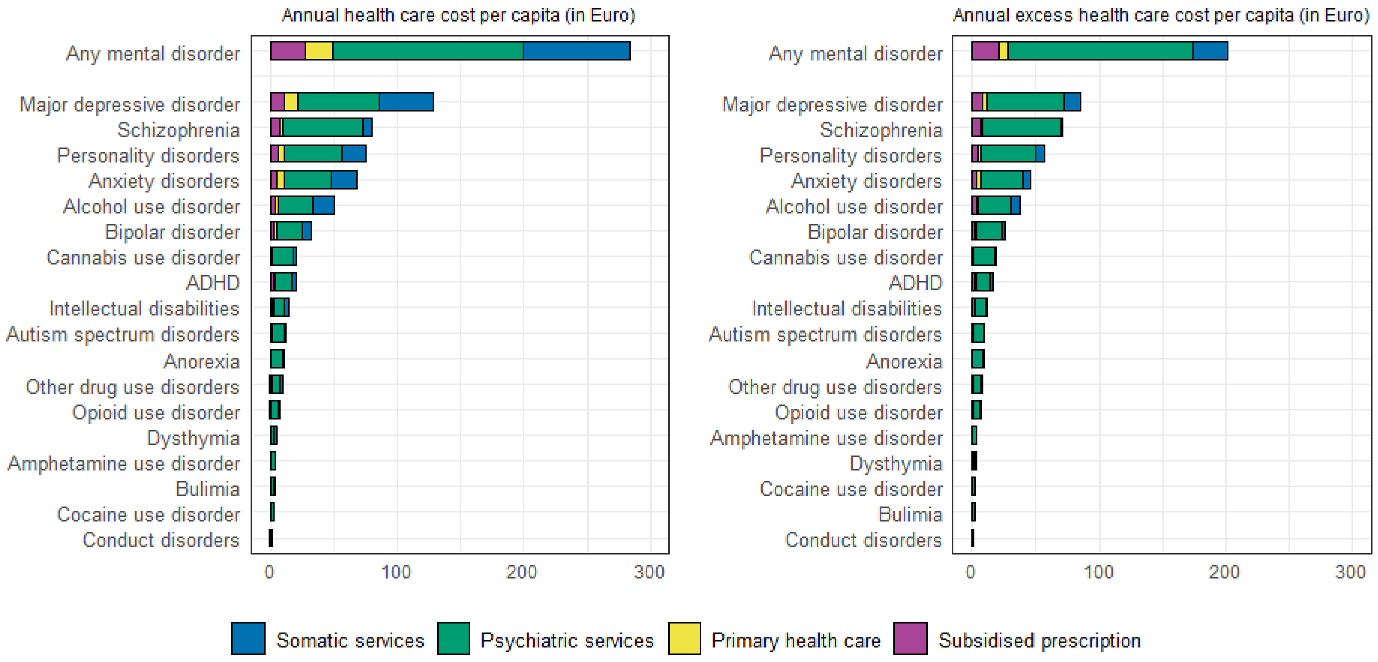

Supplementary Figure 2: Ranked nationwide annual health care cost (Euro 2017) for 18 mental disorder types by sex. The share of the different cost categories are indicated by the colours.

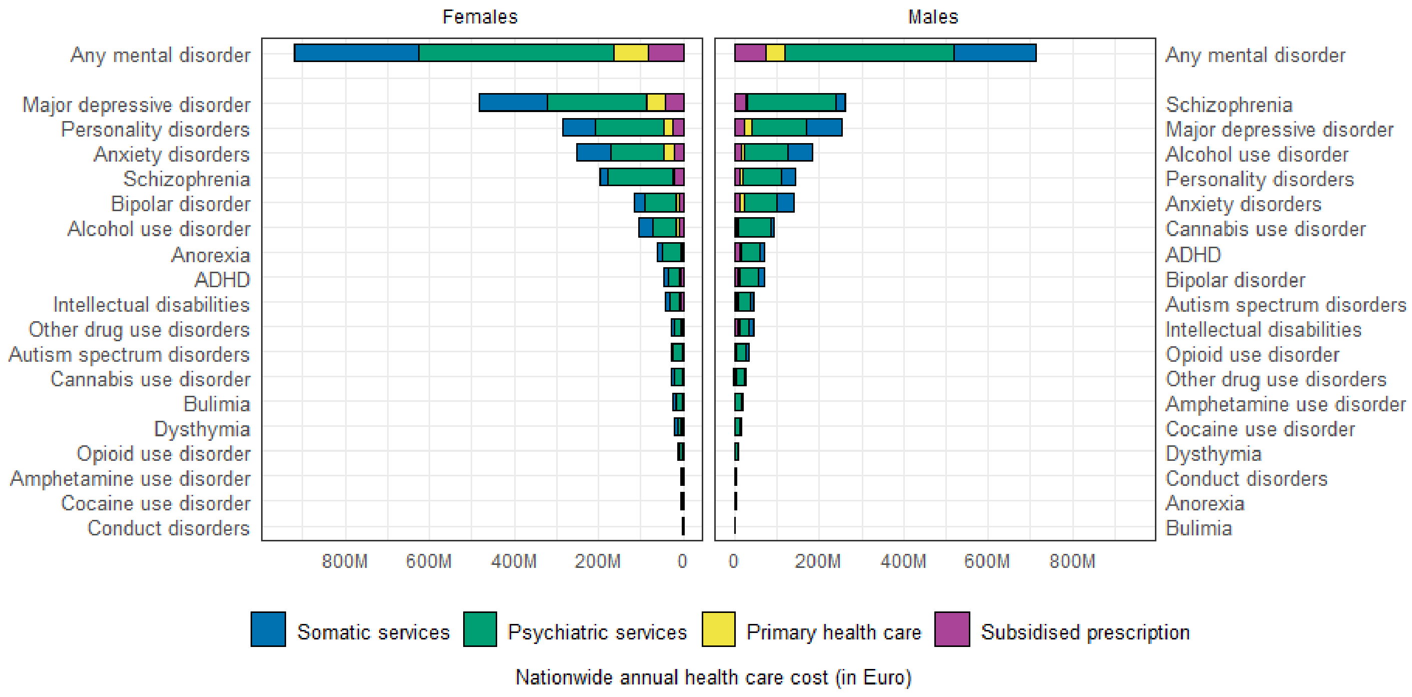

Supplementary Figure 3: Ranked nationwide annual excess health care cost (Euro 2017) for 18 mental disorder types by sex. The share of the different cost categories are indicated by the colours.

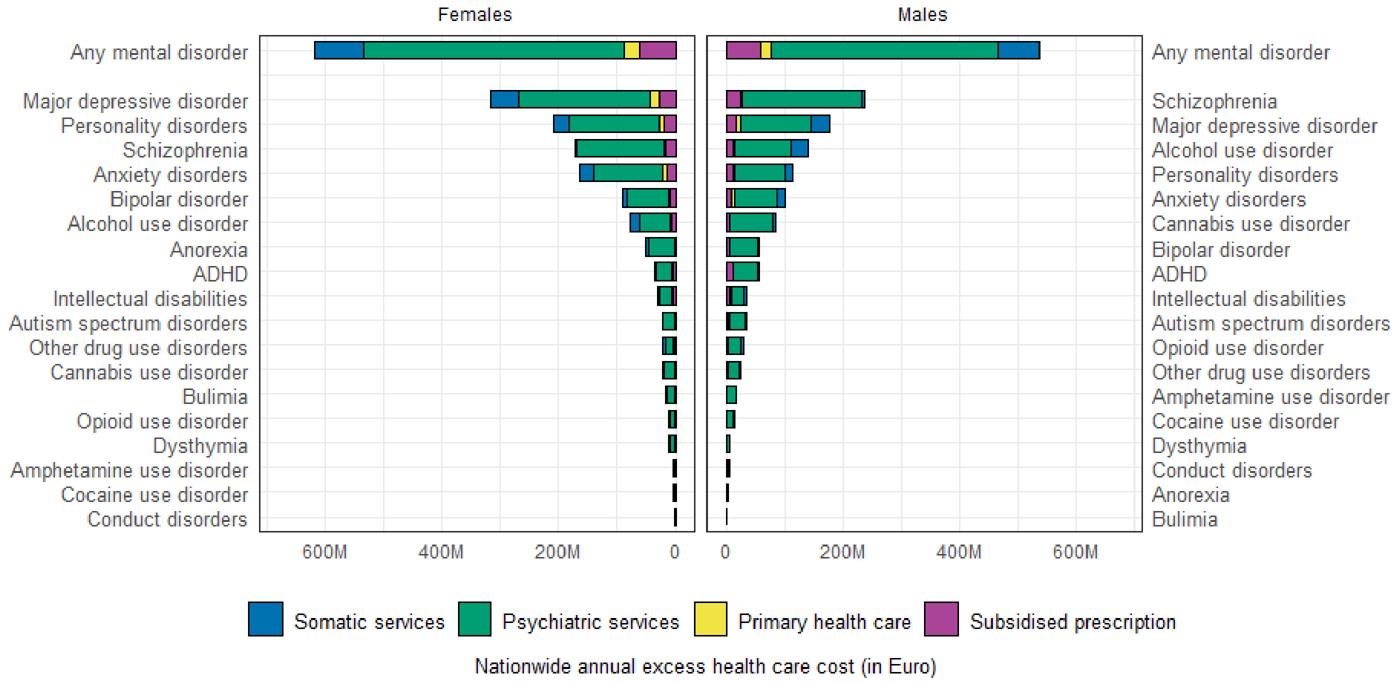

Supplementary Figure 4: Ranked annual health care cost per case (Euro 2017) for 18 mental disorder types by sex. The share of the different cost categories are indicated by the colours.

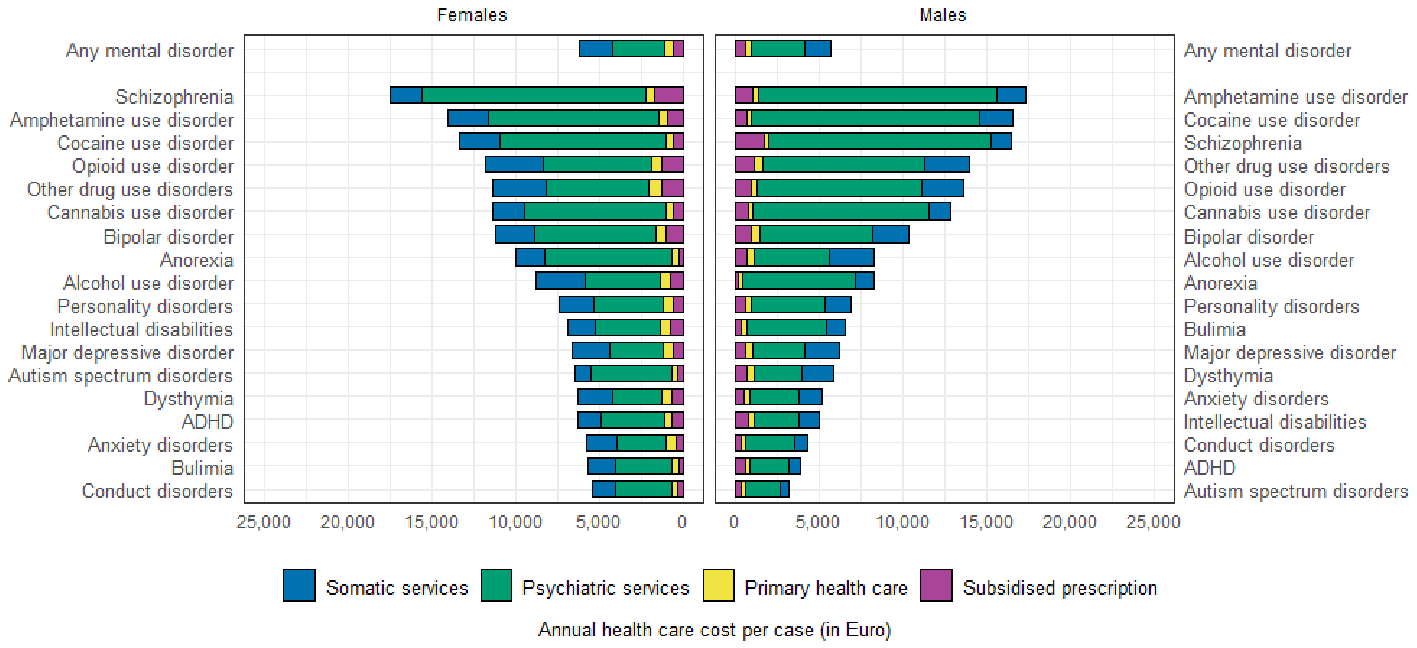

Supplementary Figure 5: Ranked annual excess health care cost per case (Euro 2017) for 18 mental disorder types by sex. The share of the different cost categories are indicated by the colours.

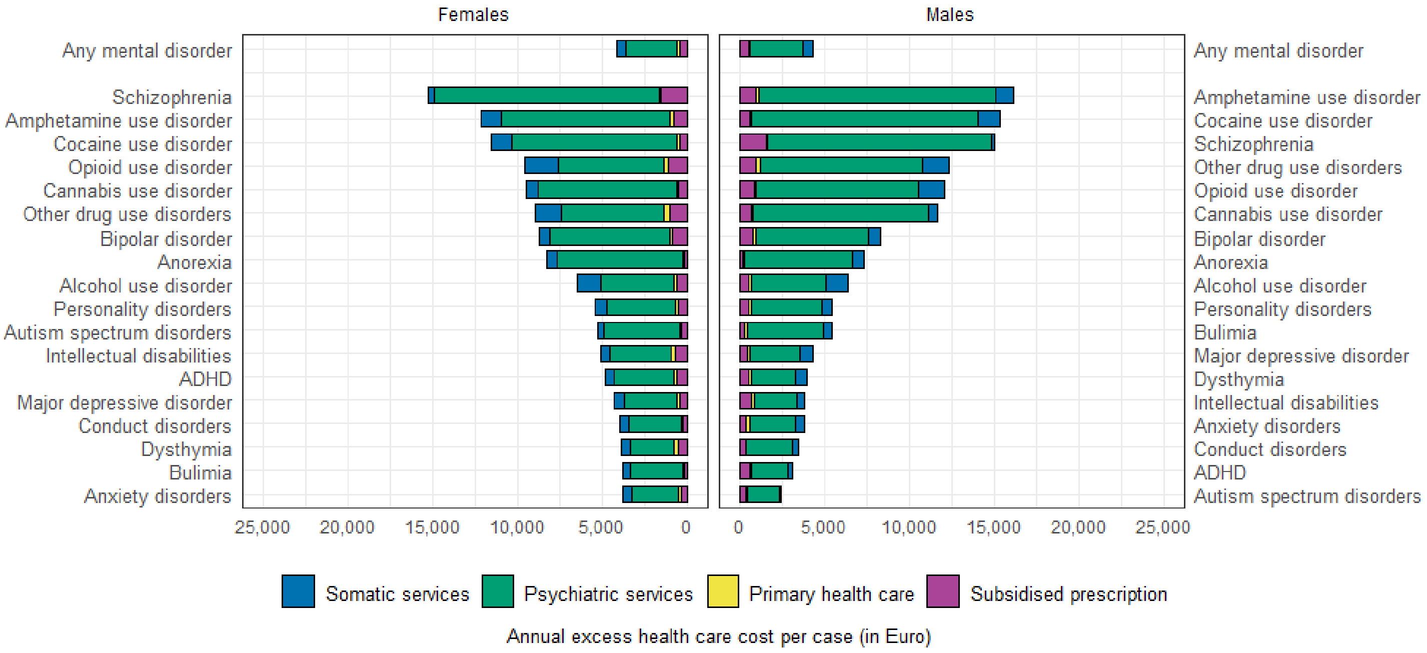

Supplementary Figure 6: Ranked annual health care cost per capita (Euro 2017) for 18 mental disorder types by sex. The share of the different cost categories are indicated by the colours.

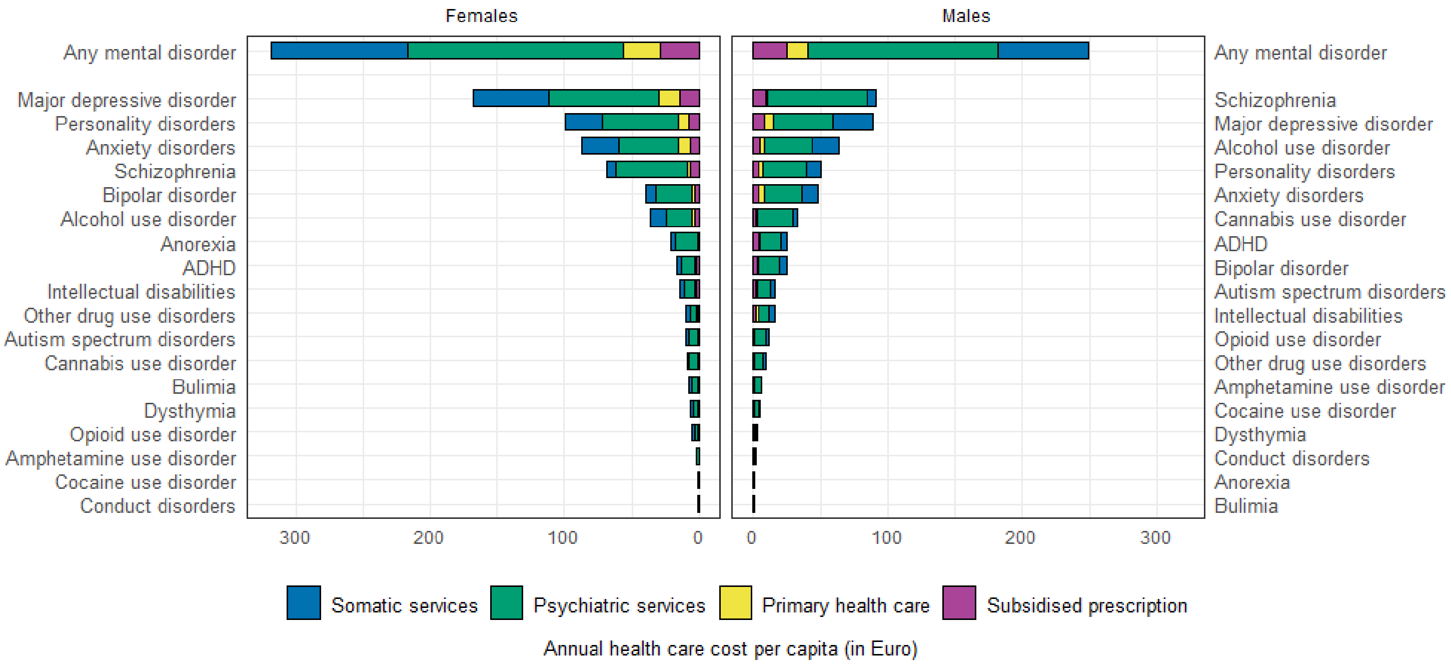

Supplementary Figure 7: Ranked annual excess health care cost per capita (Euro 2017) for 18 mental disorder types by sex. The share of the different cost categories are indicated by the colours.

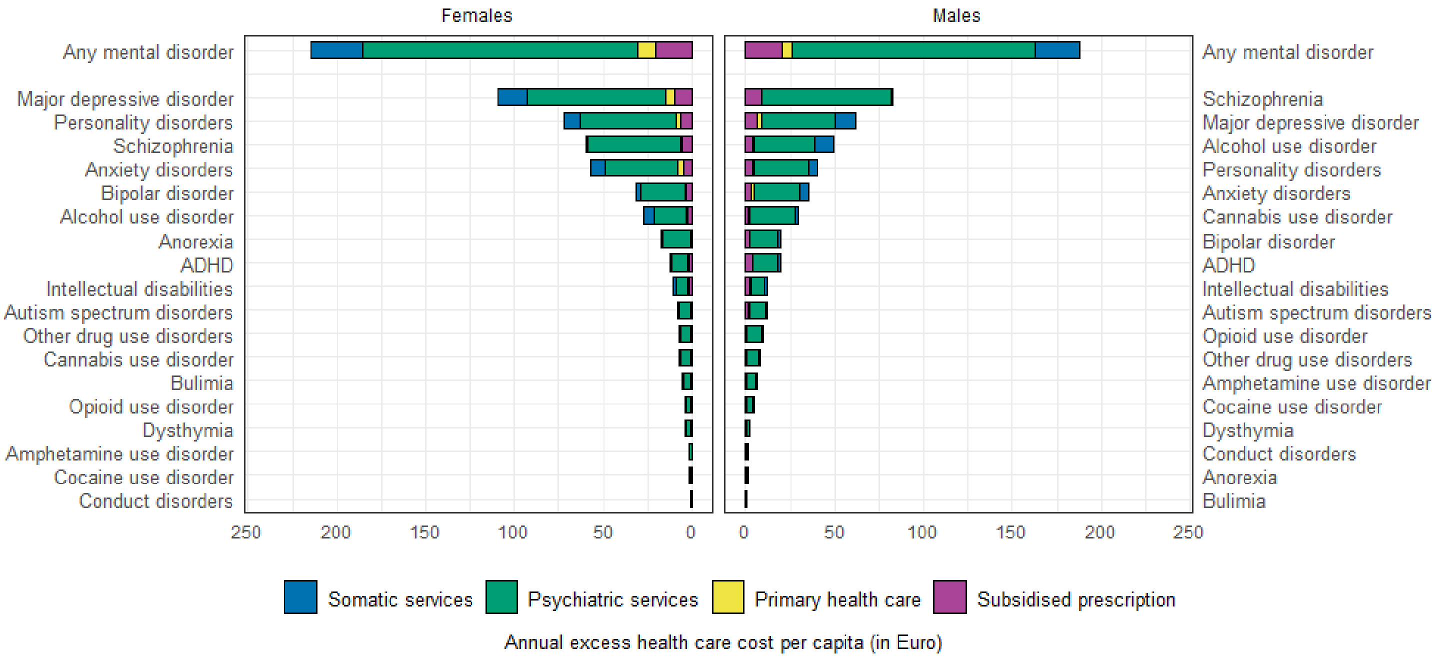

Supplementary Figure 8: Ranked annual income loss per capita (Euro 2017) for 18 mental disorder types. The different categories are indicated by the colours.

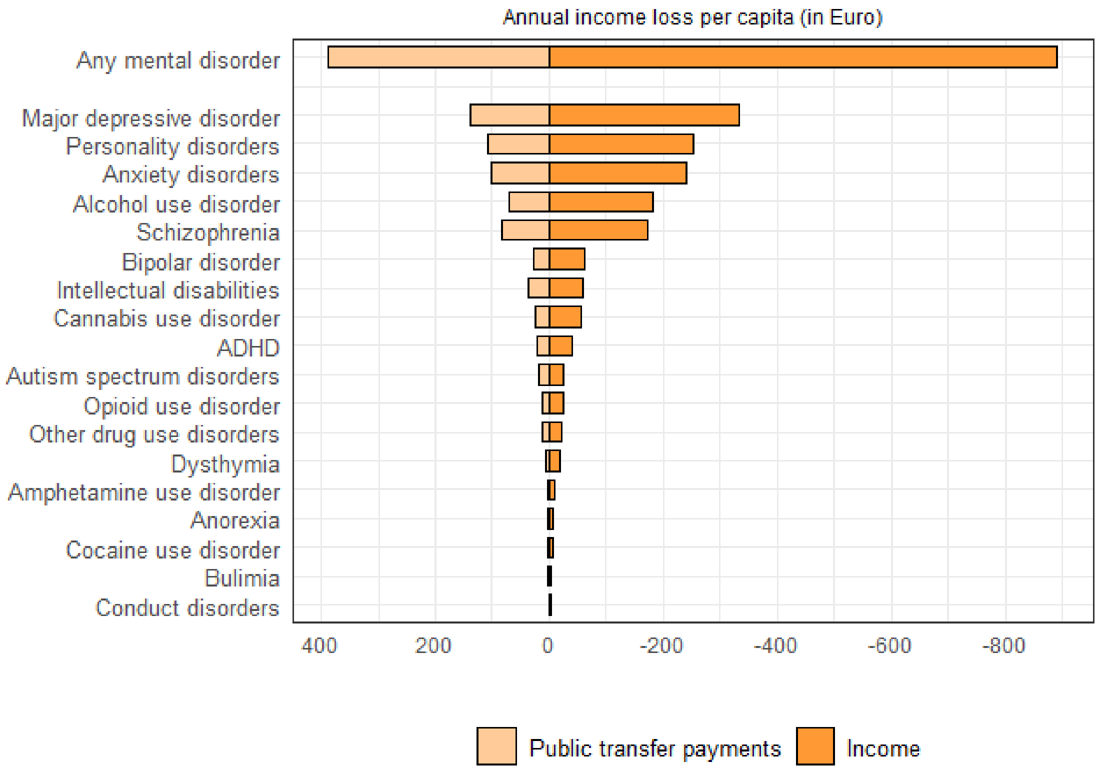

Supplementary Figure 9: Ranked nationwide annual income loss (Euro 2017) for 18 mental disorder types by sex. The different categories are indicated by the colours.

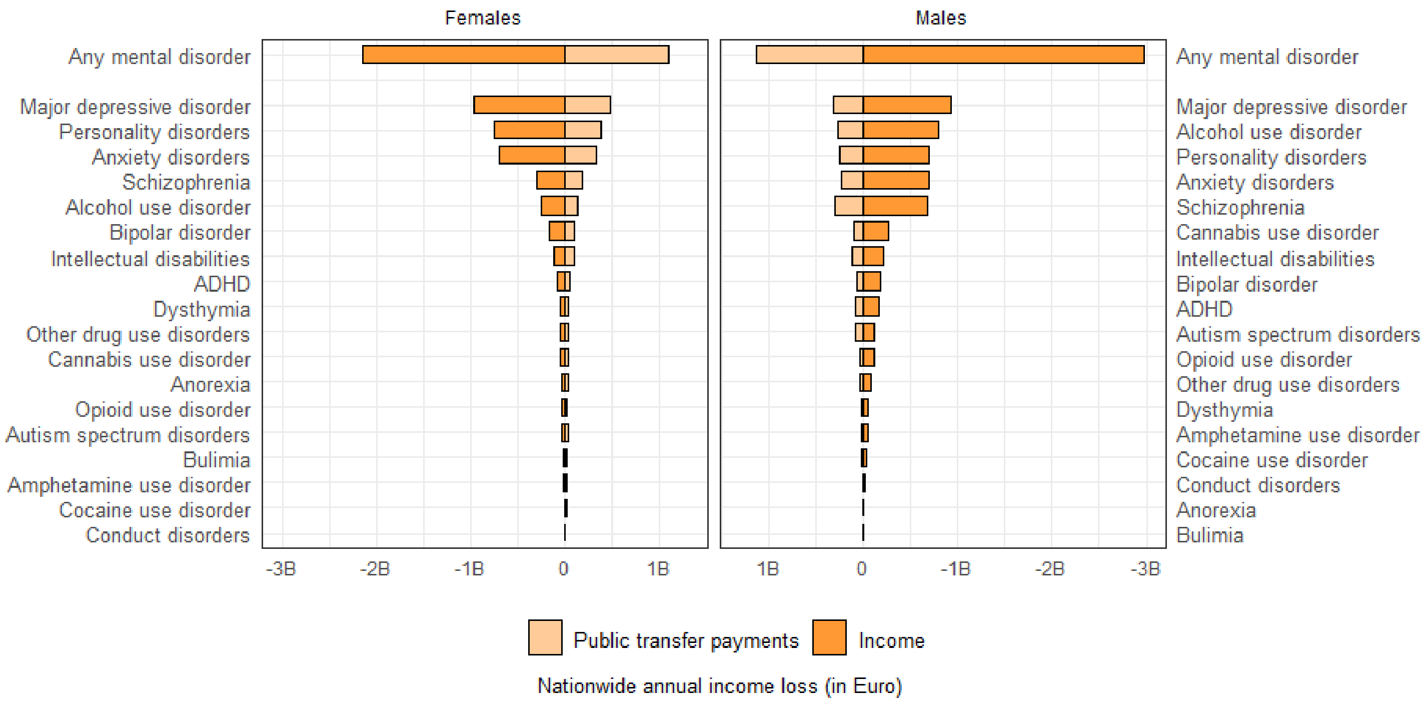

Supplementary Figure 10: Ranked annual income loss per case (Euro 2017) for 18 mental disorder types by sex. The different categories are indicated by the colours.

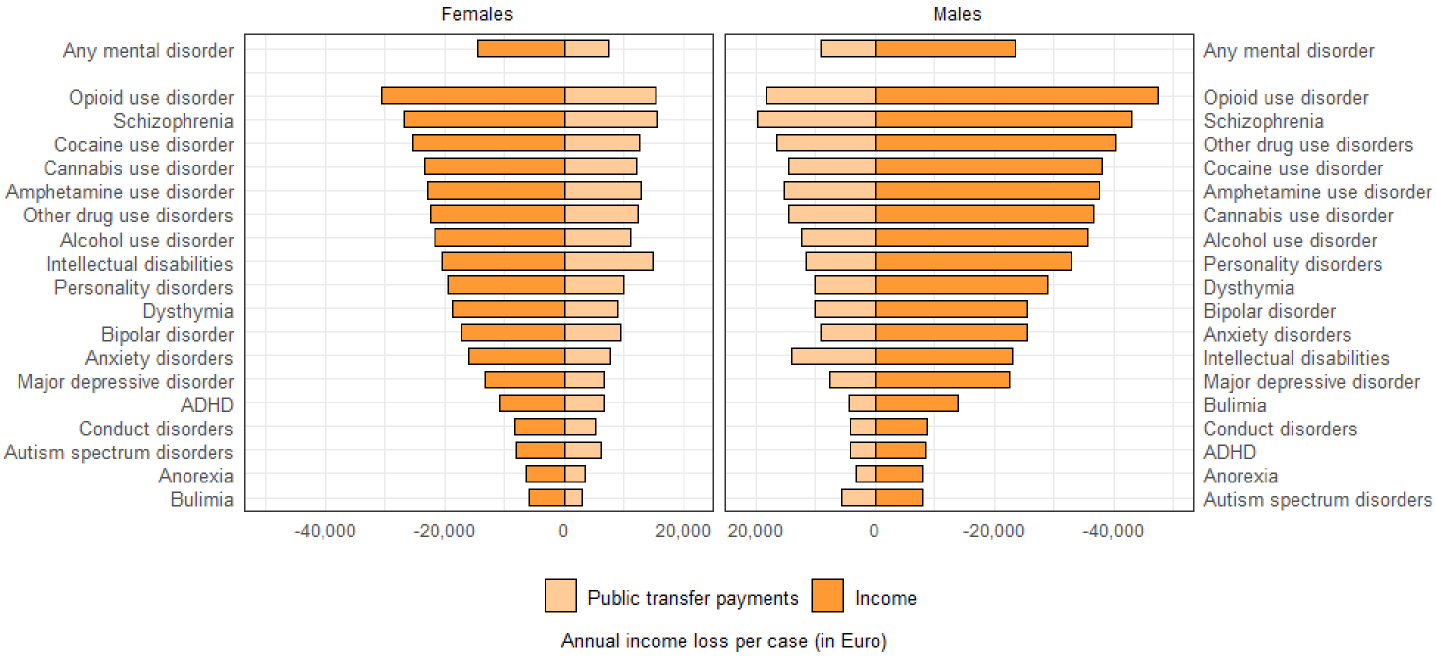

Supplementary Figure 11: Ranked annual income loss per capita (Euro 2017) for 18 mental disorder types by sex. The different categories are indicated by the colours.

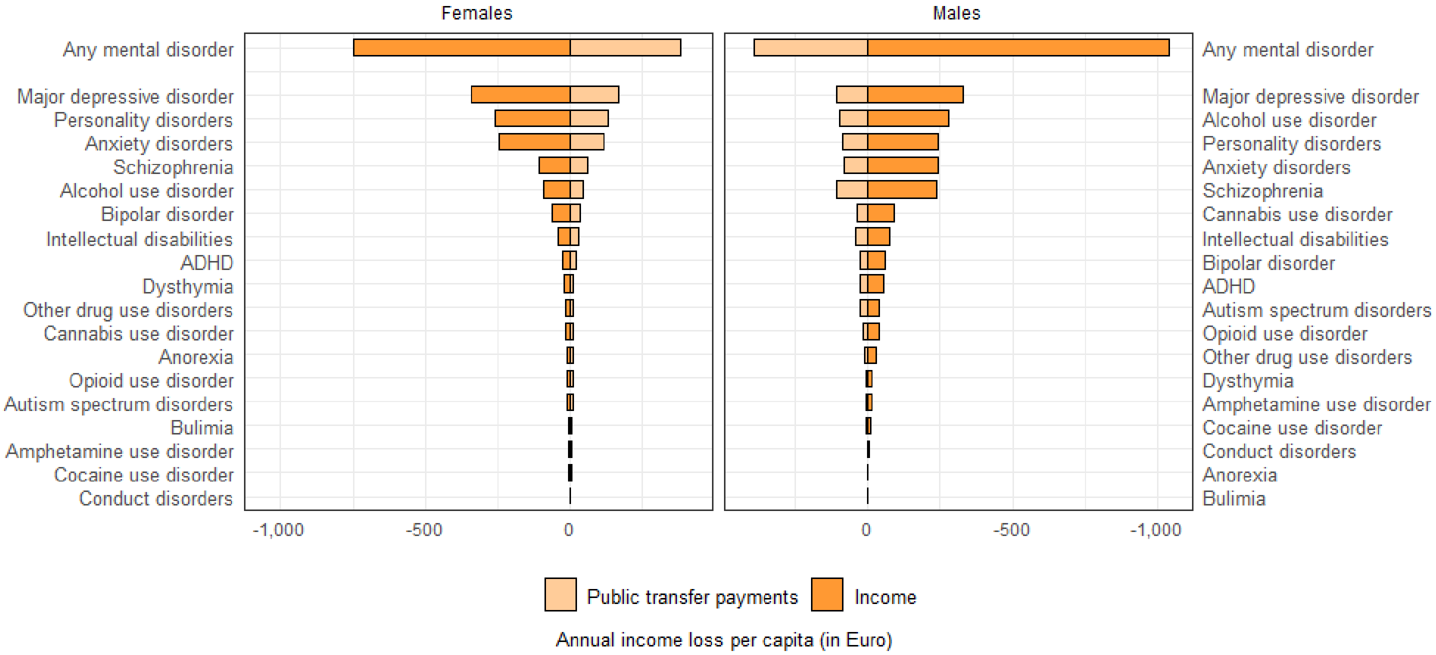

Figures by age and years after diagnosis for any mental disorder

Supplementary Figure 12: Nationwide annual health care cost and nationwide annual income loss (Euro 2017) by age and years since disorder onset.

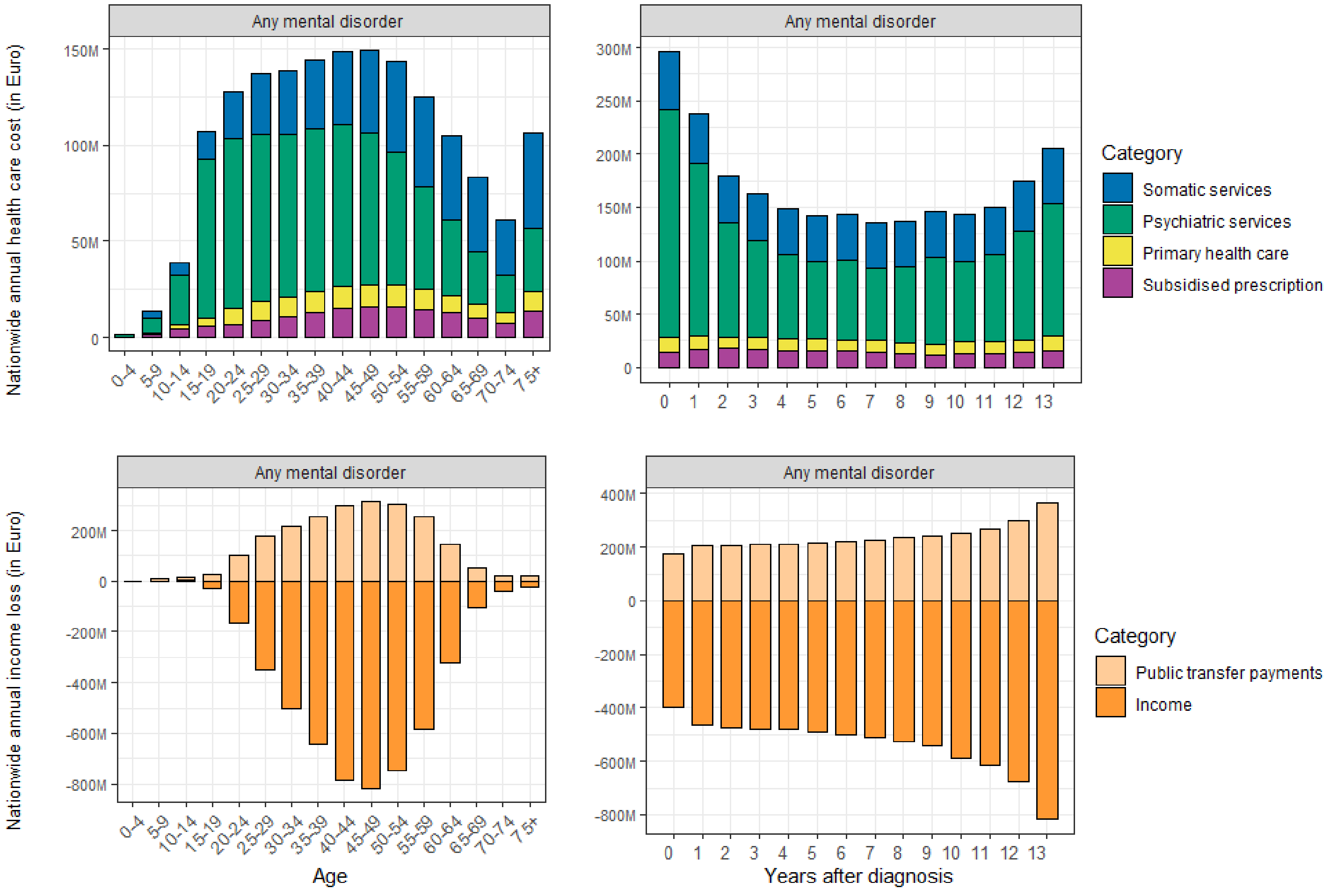

Supplementary Figure 13: Annual health care cost and annual income loss per case (Euro 2017) by age and years since disorder onset.

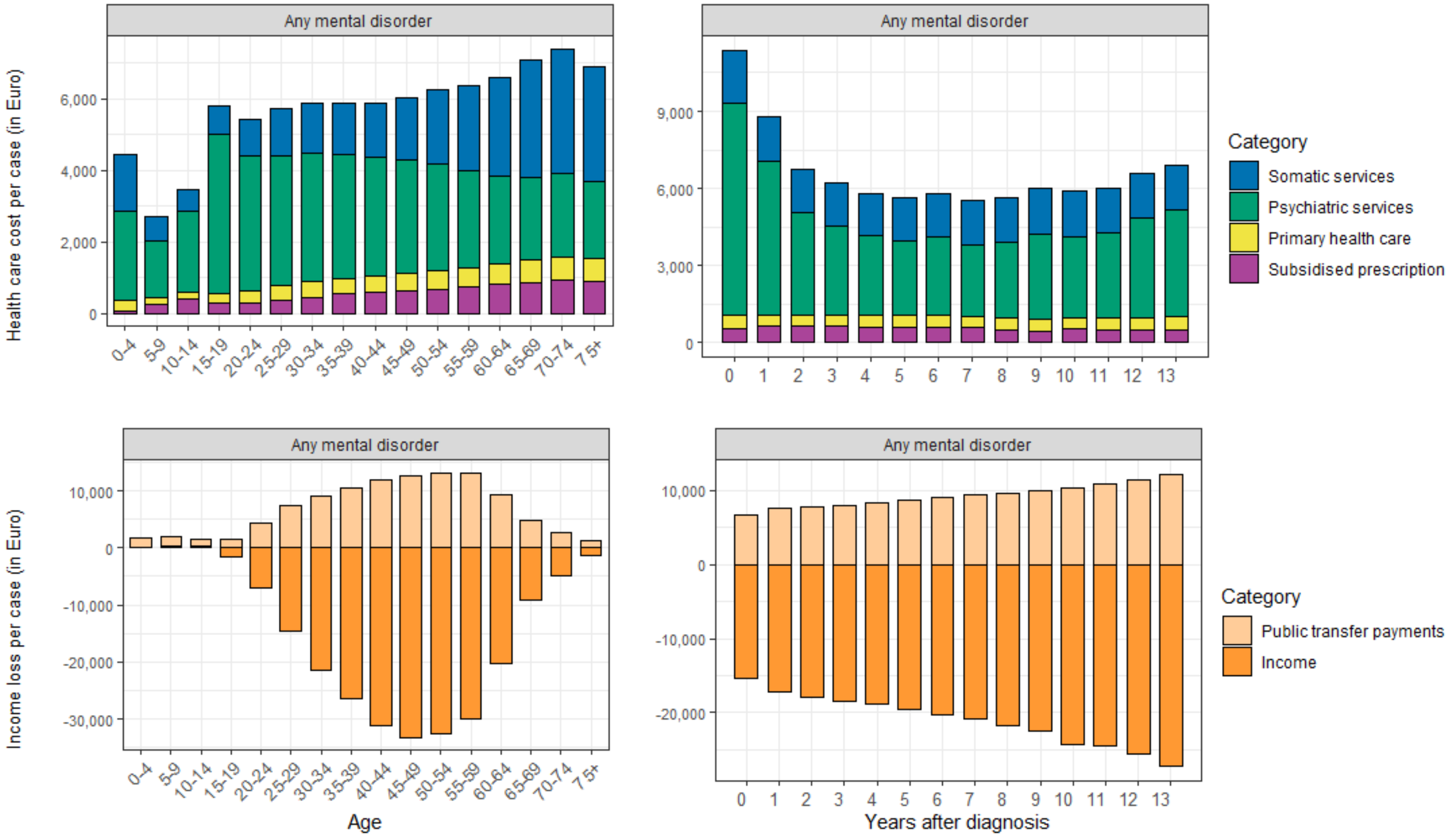

Supplementary Figure 14: Annual excess health care cost and annual income loss per case (Euro 2017) by age and years since disorder onset.

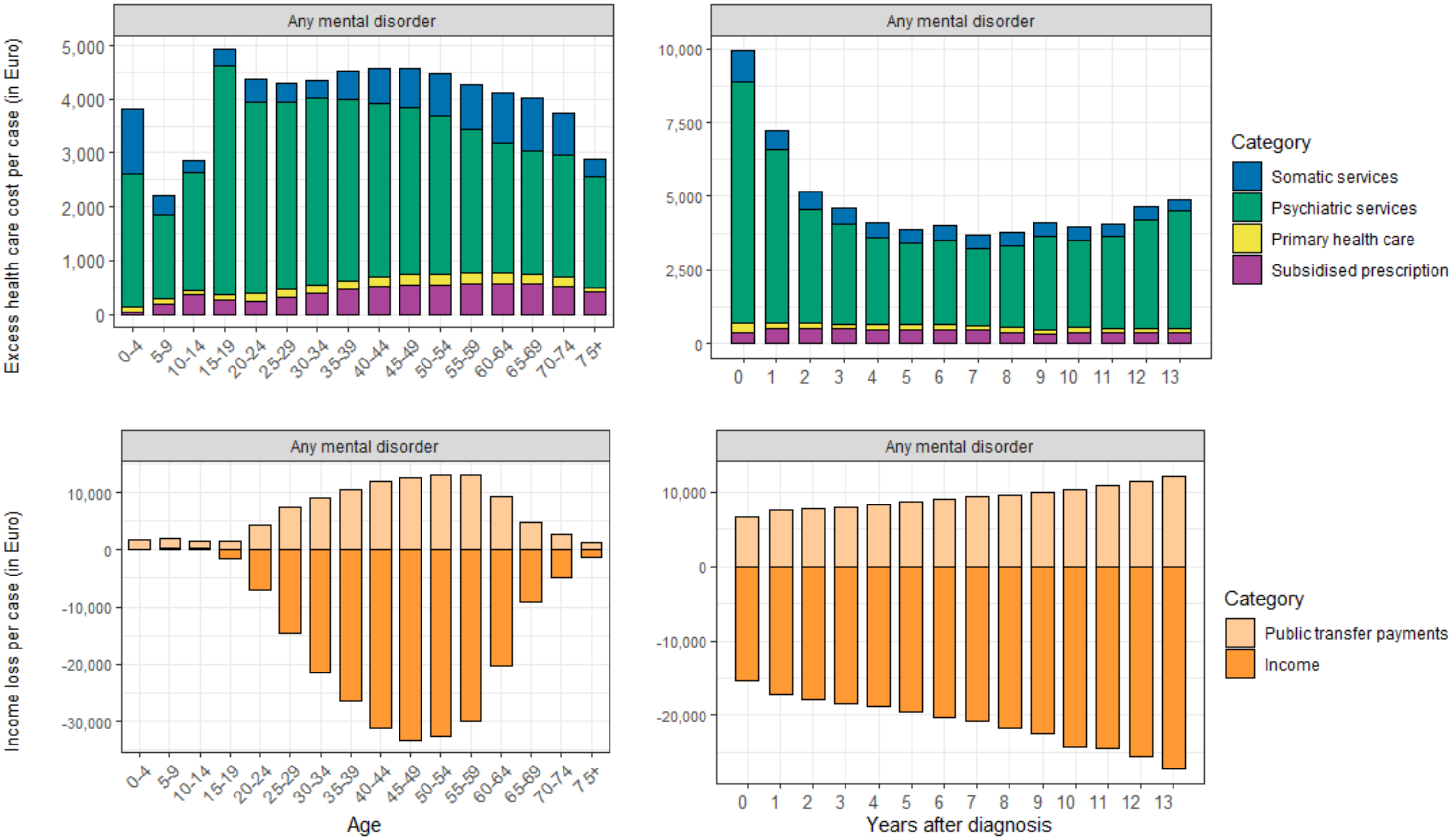

Figures by age and mental disorder type

Supplementary Figure 15 to 20

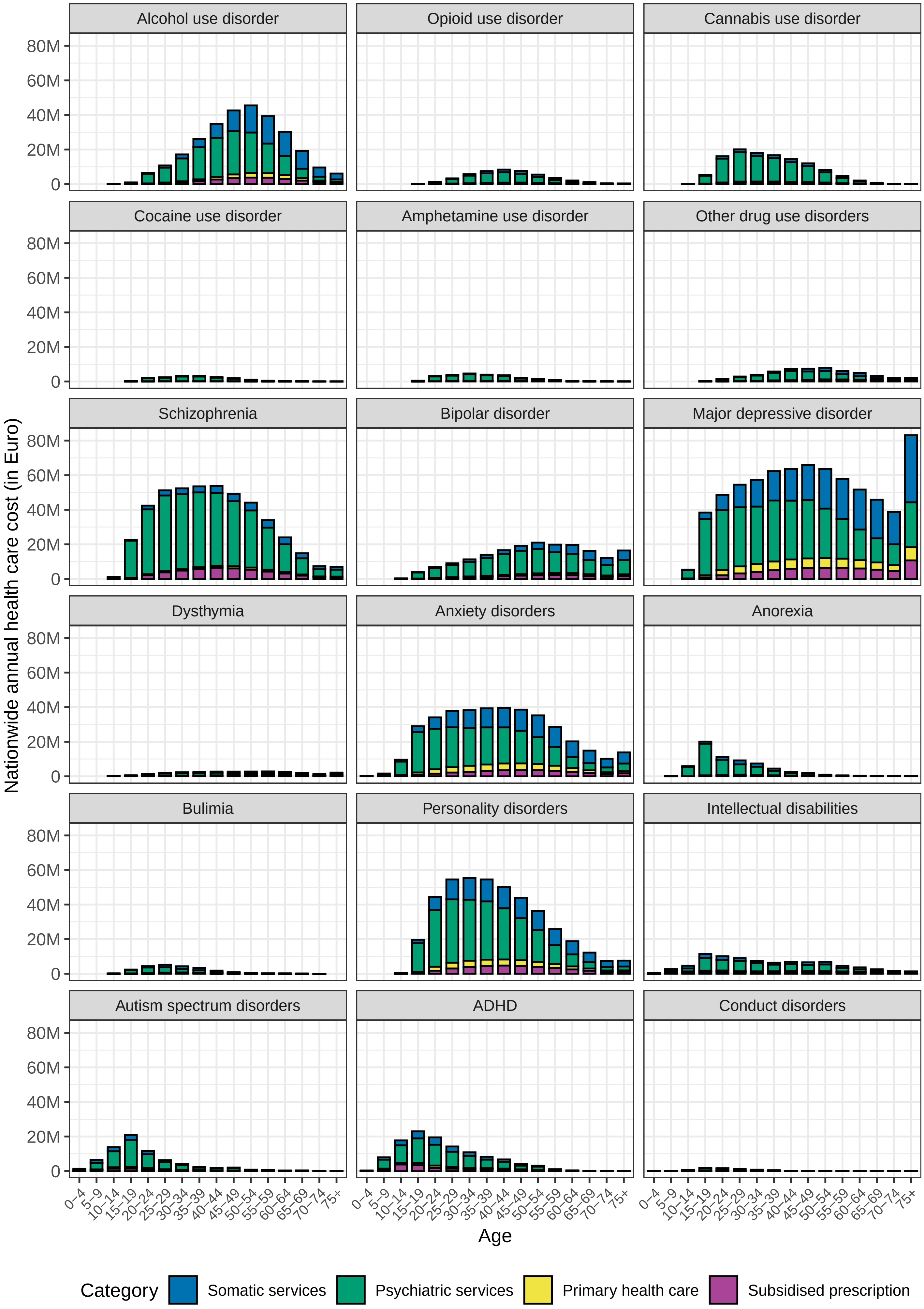

Alcohol use disorder
Opioid use disorder
Cannabis use disorder
Cocaine use disorder
Amphetamine use disorder
Other drug use disorders
Schizophrenia
Bipolar disorder
Major depressive disorder
Dysthymia
Anxiety disorders
Anorexia
Bulimia
Personality disorders
Intellectual disabilities
Autism spectrum disorders
ADHD
Conduct disorders
Nationwide annual health care cost (in Euro)
0
20M
40M
60M
80M
0–4
5–9
10–14
15–19
20–24
25–29
30–34
35–39
40–44
45–49
50–54
55–59
60–64
65–69
70–74
75+
Age
Category
Somatic services
Psychiatric services
Primary health care
Subsidised prescription

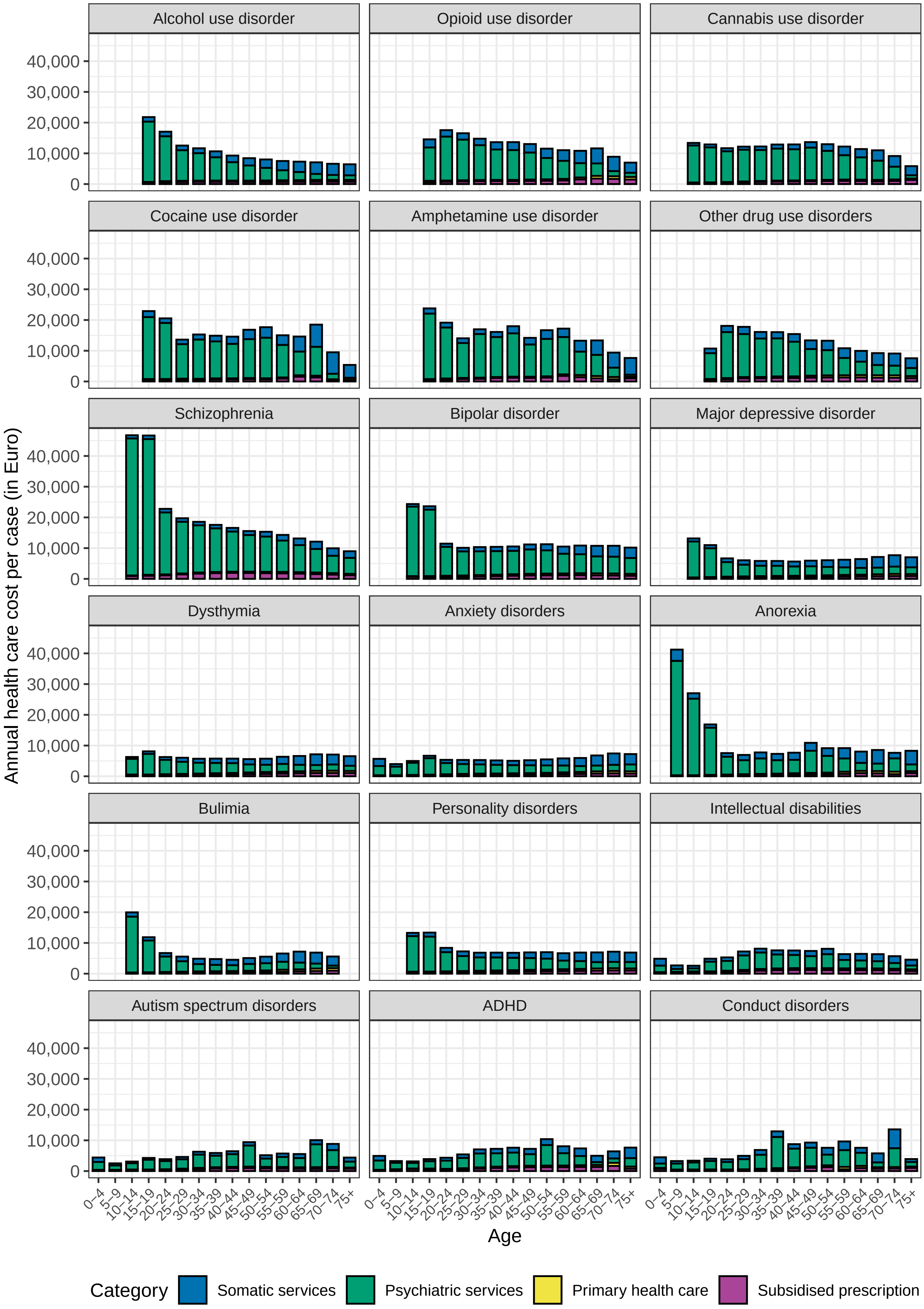

Alcohol use disorder
Opioid use disorder
Cannabis use disorder
Cocaine use disorder
Amphetamine use disorder
Other drug use disorders
Schizophrenia
Bipolar disorder
Major depressive disorder
Dysthymia
Anxiety disorders
Anorexia
Bulimia
Personality disorders
Intellectual disabilities
Autism spectrum disorders
ADHD
Conduct disorders
Annual health care cost per case (in Euro)
0
10,000
20,000
30,000
40,000
Age
0–4
5–9
10–14
15–19
20–24
25–29
30–34
35–39
40–44
45–49
50–54
55–59
60–64
65–69
70–74
75+
Category
Somatic services
Psychiatric services
Primary health care
Subsidised prescription

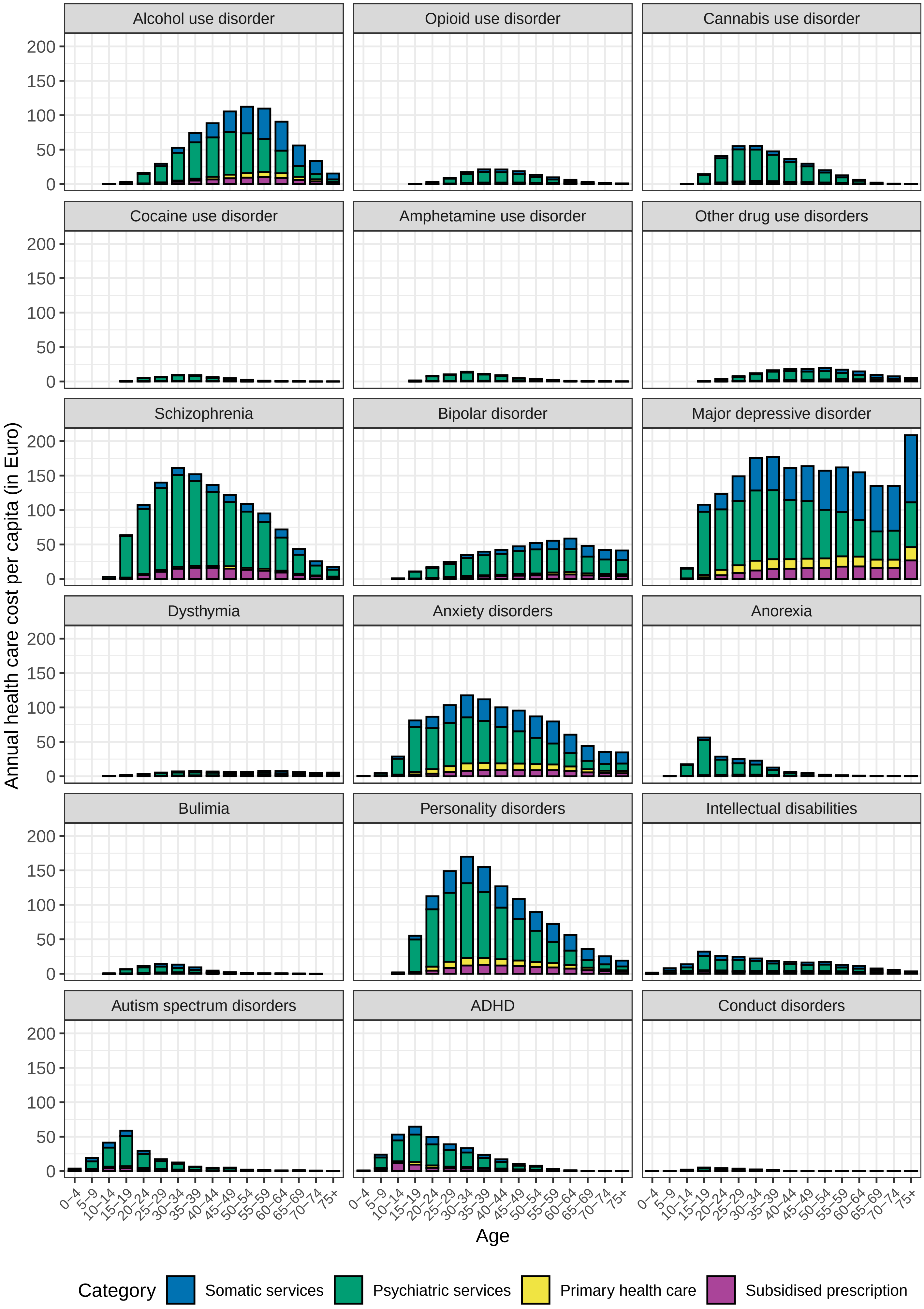
Alcohol use disorder
Opioid use disorder
Cannabis use disorder
Cocaine use disorder
Amphetamine use disorder
Other drug use disorders
Schizophrenia
Bipolar disorder
Major depressive disorder
Dysthymia
Anxiety disorders
Anorexia
Bulimia
Personality disorders
Intellectual disabilities
Autism spectrum disorders
ADHD
Conduct disorders
Annual health care cost per capita (in Euro)
0
50
100
150
200
0–4
5–9
10–14
15–19
20–24
25–29
30–34
35–39
40–44
45–49
50–54
55–59
60–64
65–69
70–74
75+
Age
Category
Somatic services
Psychiatric services
Primary health care
Subsidised prescription

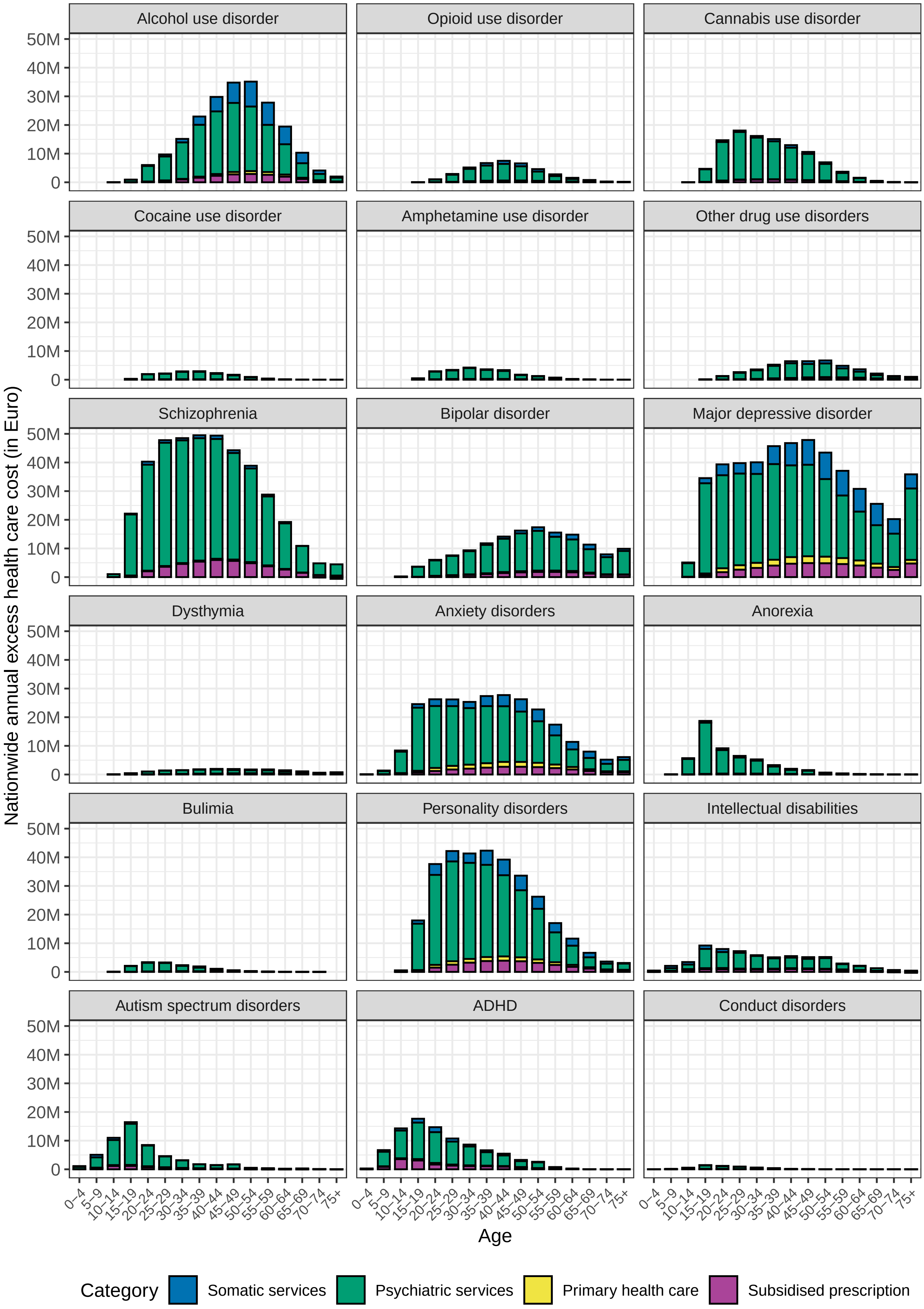
Alcohol use disorder
Opioid use disorder
Cannabis use disorder
Cocaine use disorder
Amphetamine use disorder
Other drug use disorders
Schizophrenia
Bipolar disorder
Major depressive disorder
Dysthymia
Anxiety disorders
Anorexia
Bulimia
Personality disorders
Intellectual disabilities
Autism spectrum disorders
ADHD
Conduct disorders
0
10M
20M
30M
40M
50M
Nationwide annual excess health care cost (in Euro)
0–4
5–9
10–14
15–19
20–24
25–29
30–34
35–39
40–44
45–49
50–54
55–59
60–64
65–69
70–74
75+
Age
Category
Somatic services
Psychiatric services
Primary health care
Subsidised prescription

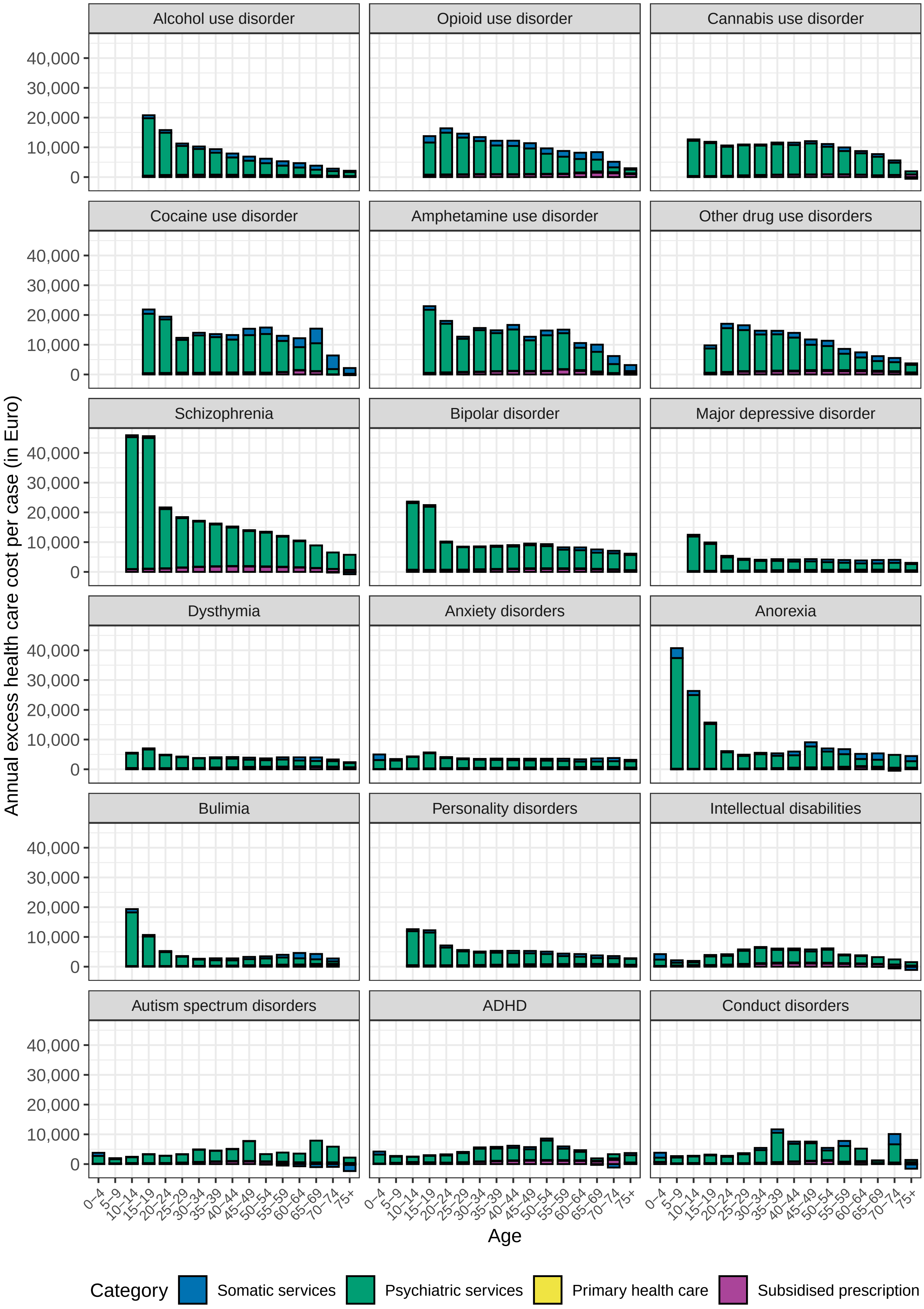

Alcohol use disorder
Opioid use disorder
Cannabis use disorder
Cocaine use disorder
Amphetamine use disorder
Other drug use disorders
Schizophrenia
Bipolar disorder
Major depressive disorder
Dysthymia
Anxiety disorders
Anorexia
Bulimia
Personality disorders
Intellectual disabilities
Autism spectrum disorders
ADHD
Conduct disorders
Annual excess health care cost per case (in Euro)
0
10,000
20,000
30,000
40,000
0–4
5–9
10–14
15–19
20–24
25–29
30–34
35–39
40–44
45–49
50–54
55–59
60–64
65–69
70–74
75+
Age
Category
Somatic services
Psychiatric services
Primary health care
Subsidised prescription

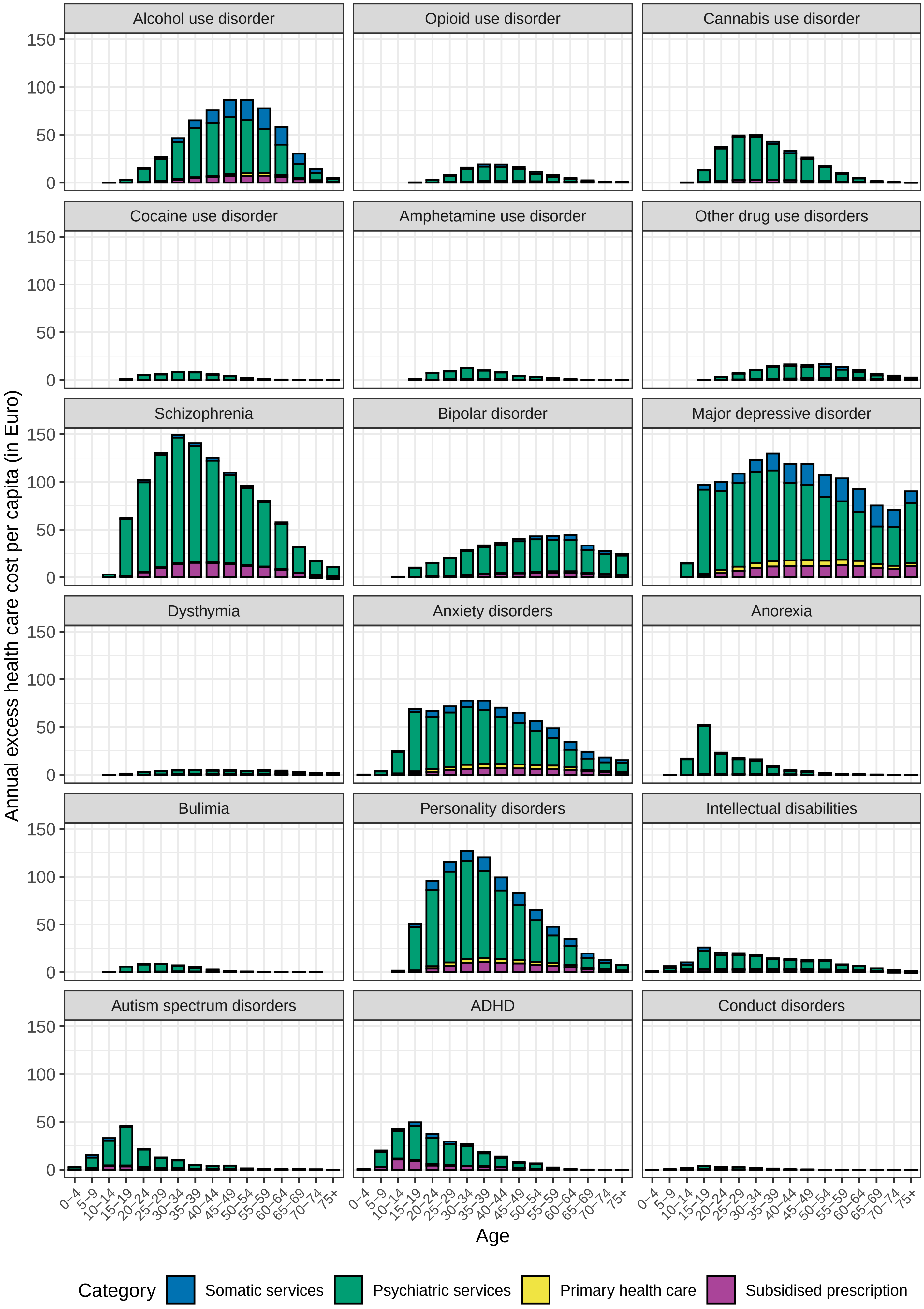

Alcohol use disorder
Opioid use disorder
Cannabis use disorder
Cocaine use disorder
Amphetamine use disorder
Other drug use disorders
Schizophrenia
Bipolar disorder
Major depressive disorder
Dysthymia
Anxiety disorders
Anorexia
Bulimia
Personality disorders
Intellectual disabilities
Autism spectrum disorders
ADHD
Conduct disorders
Annual excess health care cost per capita (in Euro)
0
50
100
150
0–4
5–9
10–14
15–19
20–24
25–29
30–34
35–39
40–44
45–49
50–54
55–59
60–64
65–69
70–74
75+
Age
Category
Somatic services
Psychiatric services
Primary health care
Subsidised prescription

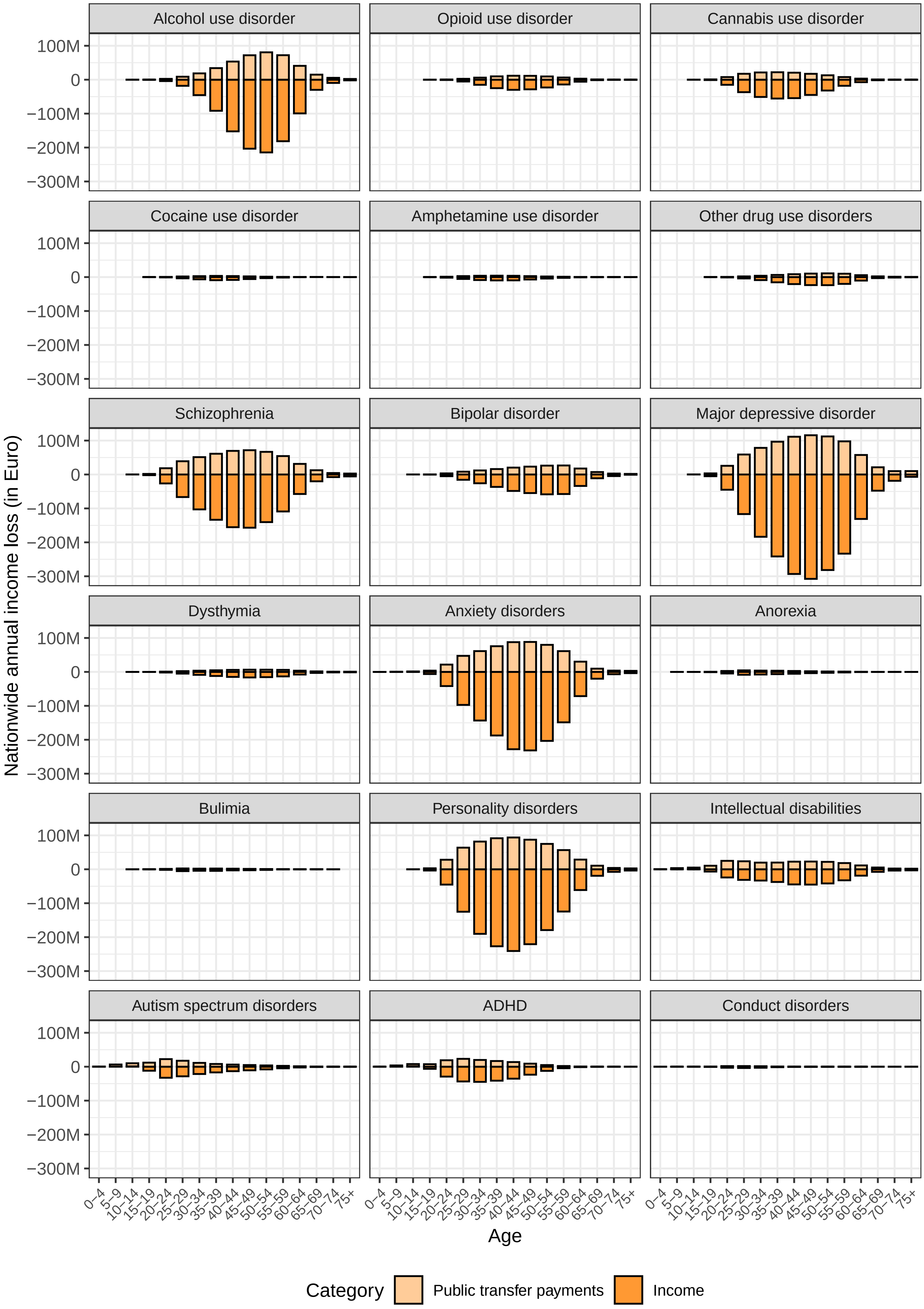

Alcohol use disorder
Opioid use disorder
Cannabis use disorder
Cocaine use disorder
Amphetamine use disorder
Other drug use disorders
Schizophrenia
Bipolar disorder
Major depressive disorder
Dysthymia
Anxiety disorders
Anorexia
Bulimia
Personality disorders
Intellectual disabilities
Autism spectrum disorders
ADHD
Conduct disorders
100M
0
−100M
−200M
−300M
Nationwide annual income loss (in Euro)
0–4
5–9
10–14
15–19
20–24
25–29
30–34
35–39
40–44
45–49
50–54
55–59
60–64
65–69
70–74
75+
Age
Category
Public transfer payments
Income

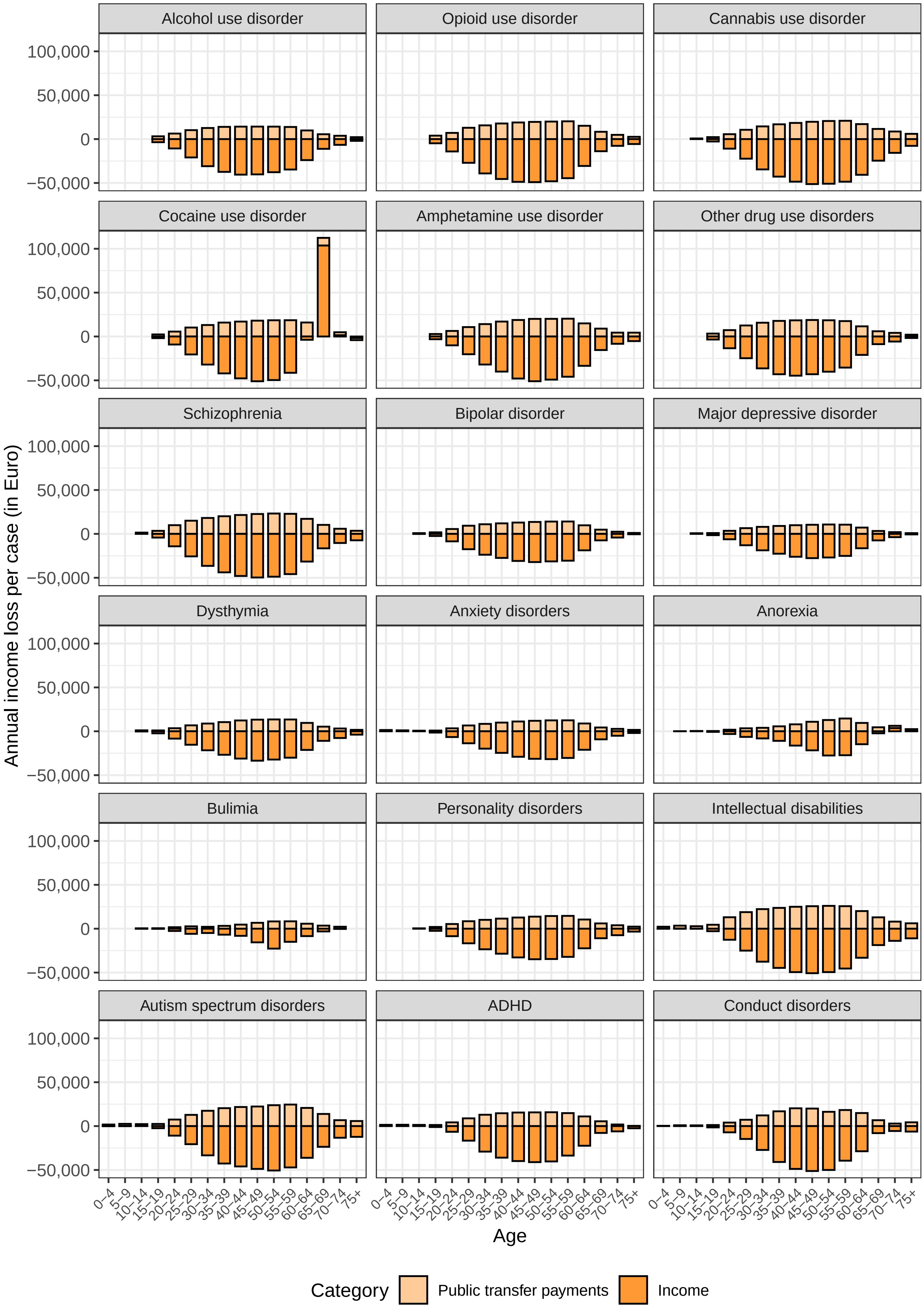

Alcohol use disorder
Opioid use disorder
Cannabis use disorder
Cocaine use disorder
Amphetamine use disorder
Other drug use disorders
Schizophrenia
Bipolar disorder
Major depressive disorder
Dysthymia
Anxiety disorders
Anorexia
Bulimia
Personality disorders
Intellectual disabilities
Autism spectrum disorders
ADHD
Conduct disorders
100,000
50,000
0
−50,000
Annual income loss per case (in Euro)
0–4
5–9
10–14
15–19
20–24
25–29
30–34
35–39
40–44
45–49
50–54
55–59
60–64
65–69
70–74
75+
Age
Category
Public transfer payments
Income

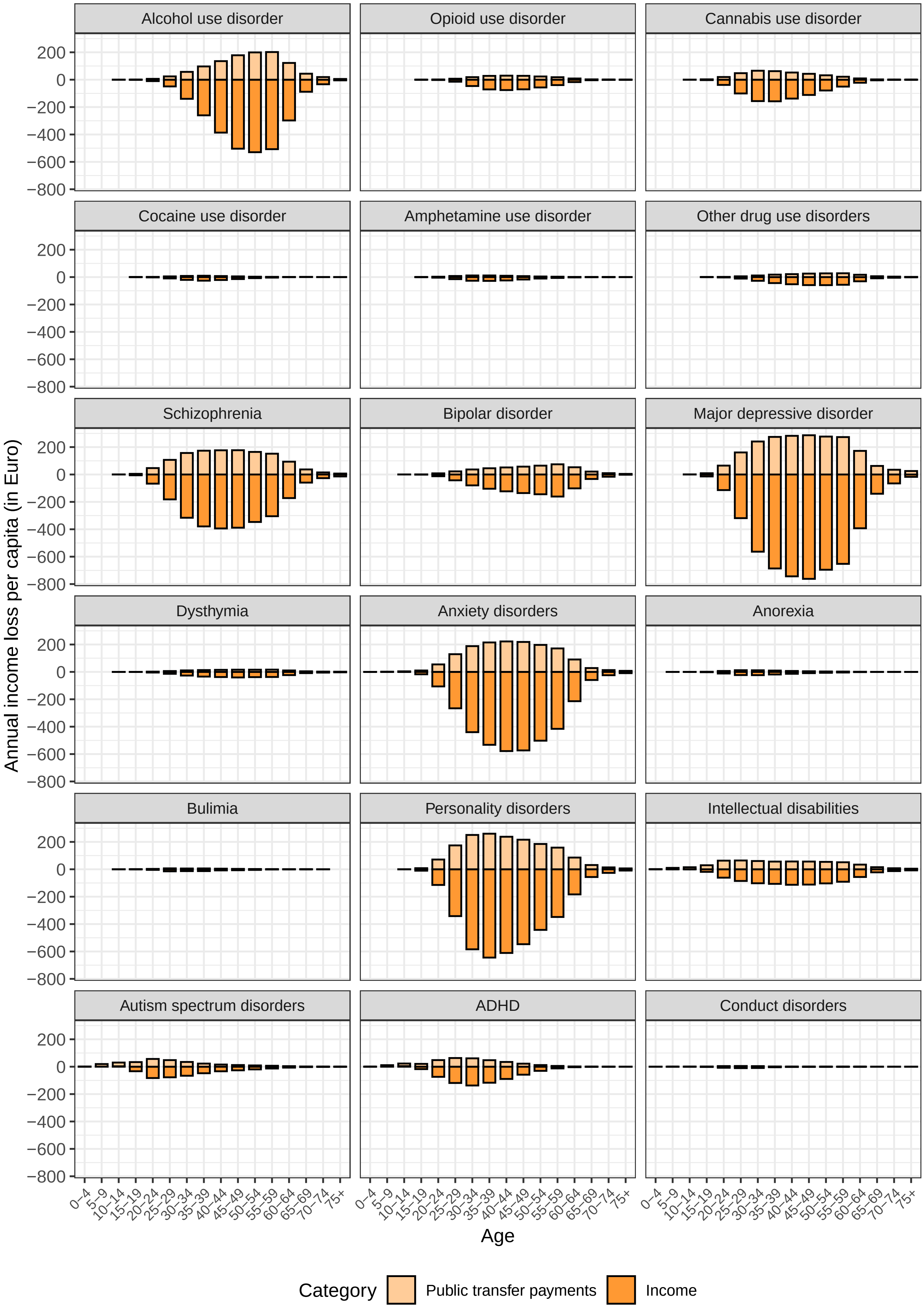
Alcohol use disorder
Opioid use disorder
Cannabis use disorder
Cocaine use disorder
Amphetamine use disorder
Other drug use disorders
Schizophrenia
Bipolar disorder
Major depressive disorder
Dysthymia
Anxiety disorders
Anorexia
Bulimia
Personality disorders
Intellectual disabilities
Autism spectrum disorders
ADHD
Conduct disorders
200
0
−200
−400
−600
−800
Annual income loss per capita (in Euro)
0–4
5–9
10–14
15–19
20–24
25–29
30–34
35–39
40–44
45–49
50–54
55–59
60–64
65–69
70–74
75+
Age
Category
Public transfer payments
Income

Figures by years after diagnosis and mental disorder type

Supplementary Figure 21 to 26

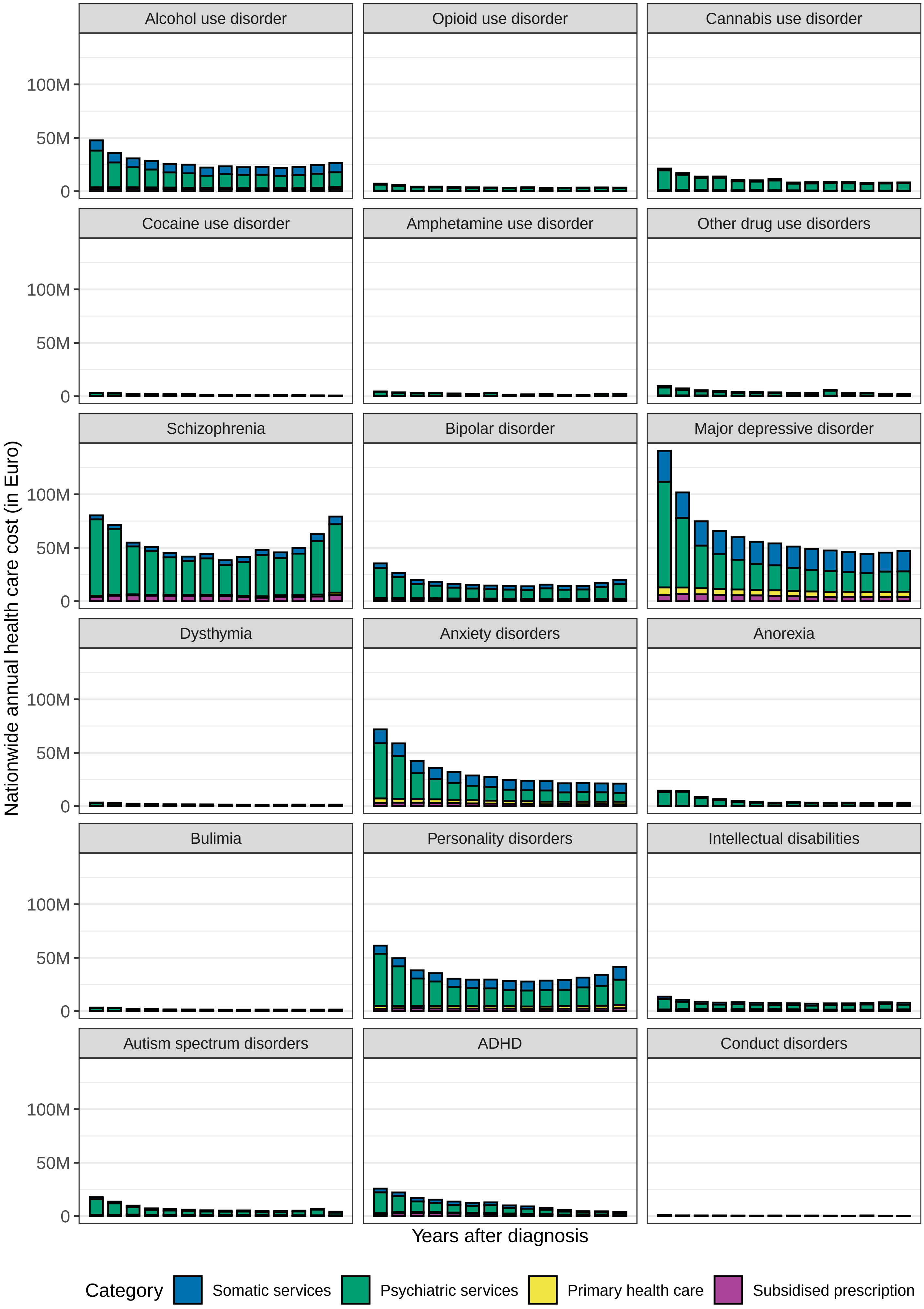

Alcohol use disorder
Opioid use disorder
Cannabis use disorder
Cocaine use disorder
Amphetamine use disorder
Other drug use disorders
Schizophrenia
Bipolar disorder
Major depressive disorder
Dysthymia
Anxiety disorders
Anorexia
Bulimia
Personality disorders
Intellectual disabilities
Autism spectrum disorders
ADHD
Conduct disorders
100M
50M
0
Nationwide annual health care cost (in Euro)
Years after diagnosis
Category
Somatic services
Psychiatric services
Primary health care
Subsidised prescription

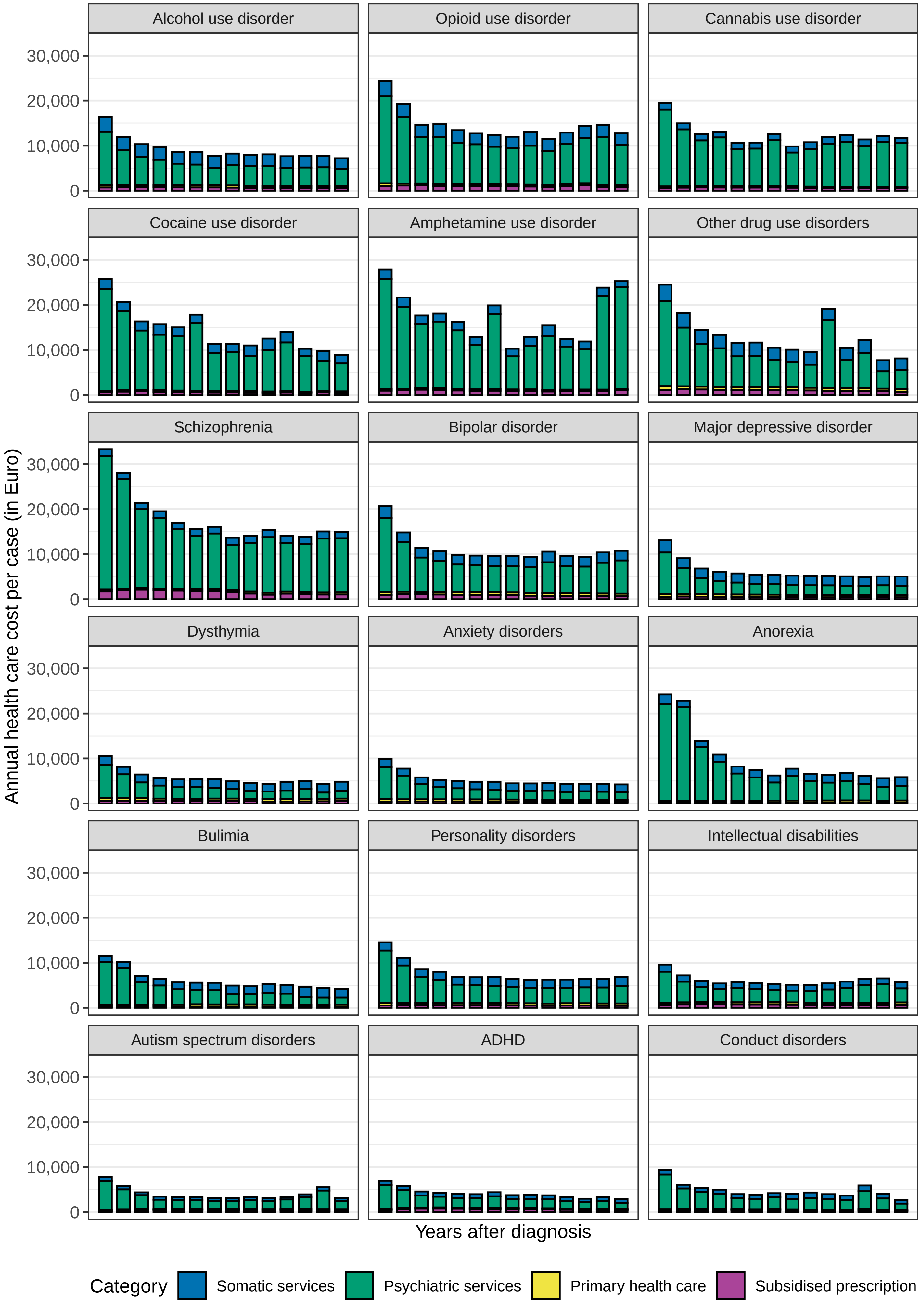

Alcohol use disorder
Opioid use disorder
Cannabis use disorder
Cocaine use disorder
Amphetamine use disorder
Other drug use disorders
Schizophrenia
Bipolar disorder
Major depressive disorder
Dysthymia
Anxiety disorders
Anorexia
Bulimia
Personality disorders
Intellectual disabilities
Autism spectrum disorders
ADHD
Conduct disorders
30,000
20,000
10,000
0
Annual health care cost per case (in Euro)
Years after diagnosis
Category
Somatic services
Psychiatric services
Primary health care
Subsidised prescription

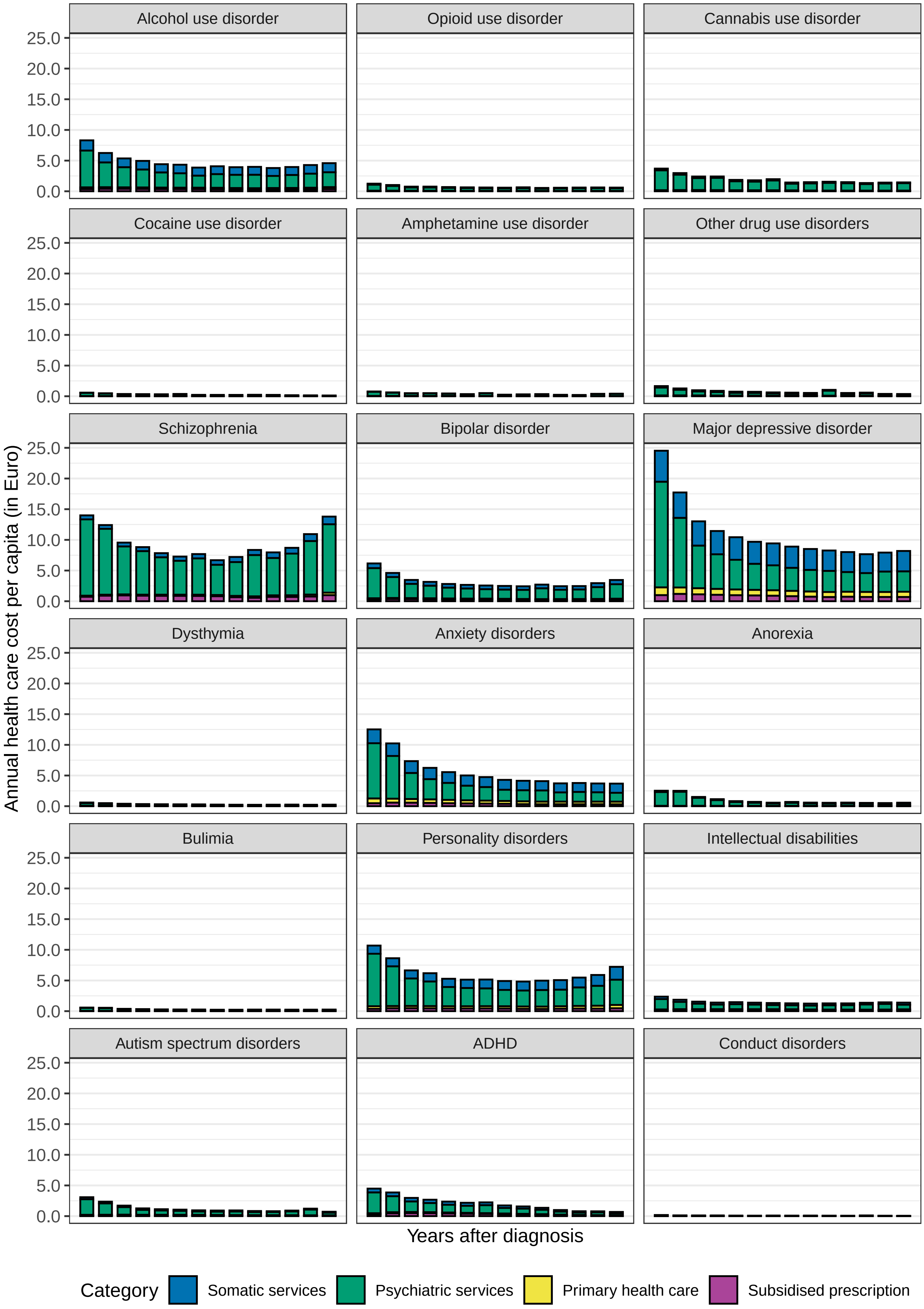
Alcohol use disorder
Opioid use disorder
Cannabis use disorder
Cocaine use disorder
Amphetamine use disorder
Other drug use disorders
Schizophrenia
Bipolar disorder
Major depressive disorder
Dysthymia
Anxiety disorders
Anorexia
Bulimia
Personality disorders
Intellectual disabilities
Autism spectrum disorders
ADHD
Conduct disorders
0.0
5.0
10.0
15.0
20.0
25.0
Annual health care cost per capita (in Euro)
Years after diagnosis
Category
Somatic services
Psychiatric services
Primary health care
Subsidised prescription

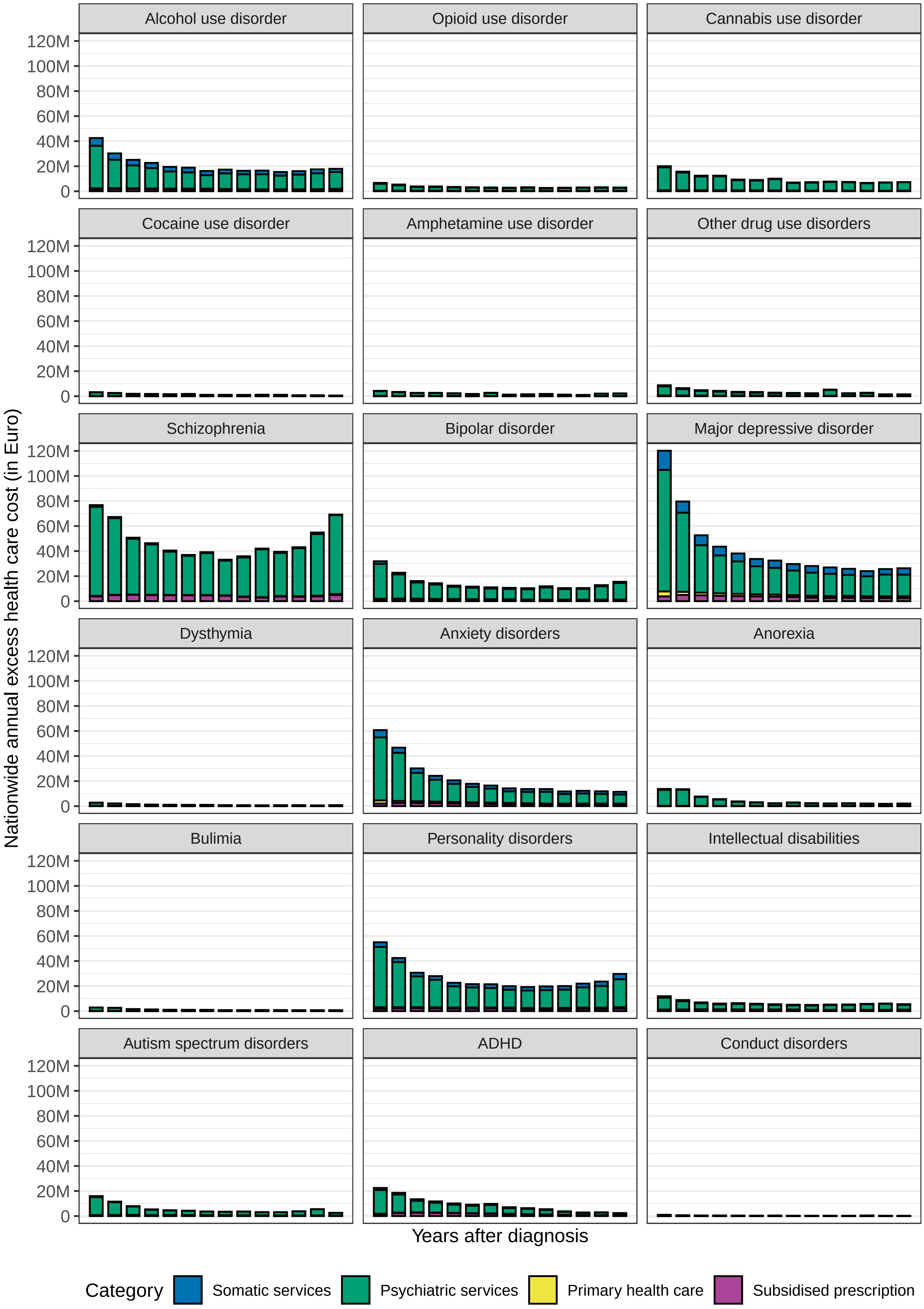

Alcohol use disorder
Opioid use disorder
Cannabis use disorder
Cocaine use disorder
Amphetamine use disorder
Other drug use disorders
Schizophrenia
Bipolar disorder
Major depressive disorder
Dysthymia
Anxiety disorders
Anorexia
Bulimia
Personality disorders
Intellectual disabilities
Autism spectrum disorders
ADHD
Conduct disorders
120M
100M
80M
60M
40M
20M
0
Nationwide annual excess health care cost (in Euro)
Years after diagnosis
Category
Somatic services
Psychiatric services
Primary health care
Subsidised prescription

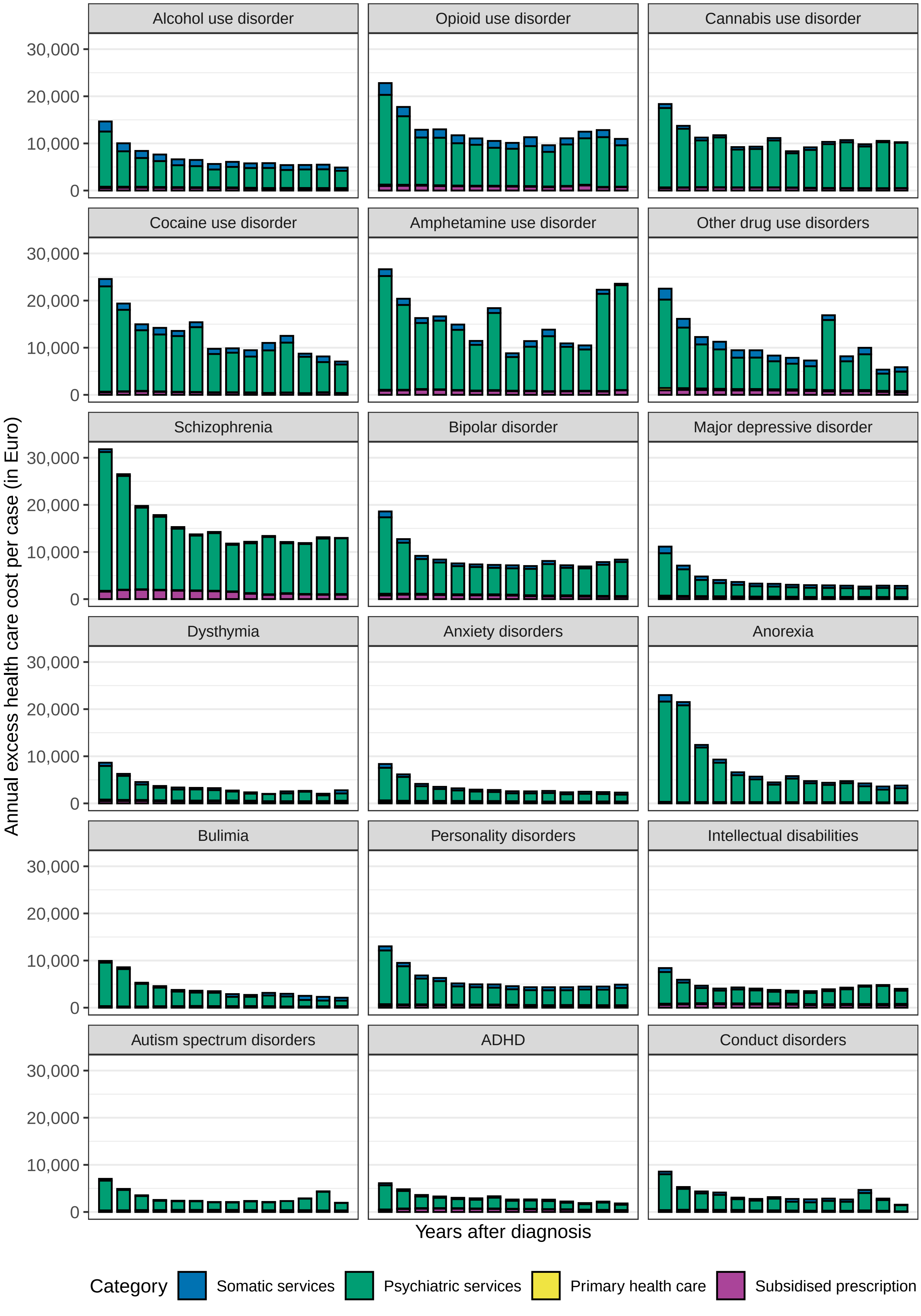

Alcohol use disorder
Opioid use disorder
Cannabis use disorder
Cocaine use disorder
Amphetamine use disorder
Other drug use disorders
Schizophrenia
Bipolar disorder
Major depressive disorder
Dysthymia
Anxiety disorders
Anorexia
Bulimia
Personality disorders
Intellectual disabilities
Autism spectrum disorders
ADHD
Conduct disorders
0
10,000
20,000
30,000
Annual excess health care cost per case (in Euro)
Years after diagnosis
Category
Somatic services
Psychiatric services
Primary health care
Subsidised prescription

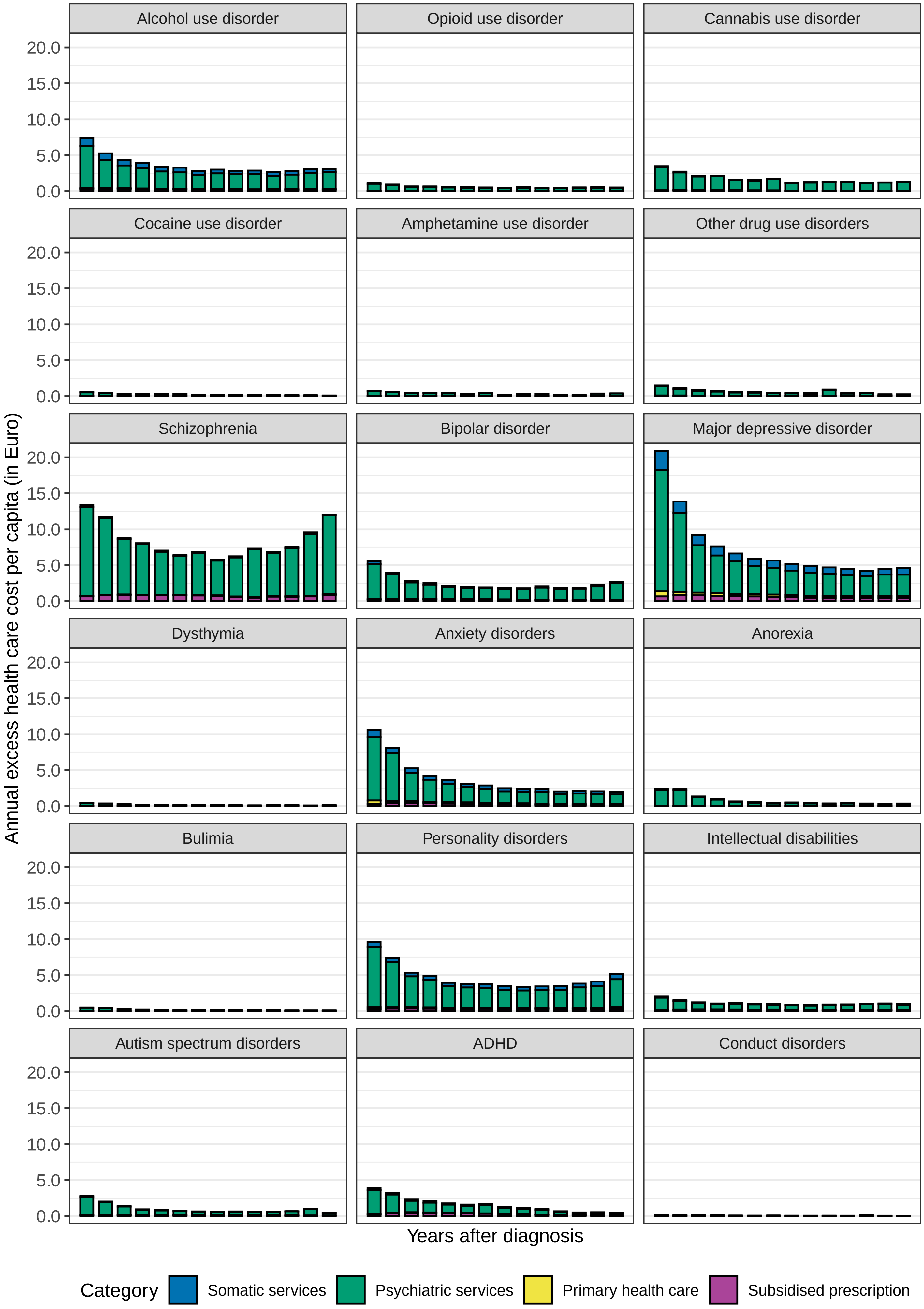
Alcohol use disorder
Opioid use disorder
Cannabis use disorder
Cocaine use disorder
Amphetamine use disorder
Other drug use disorders
Schizophrenia
Bipolar disorder
Major depressive disorder
Dysthymia
Anxiety disorders
Anorexia
Bulimia
Personality disorders
Intellectual disabilities
Autism spectrum disorders
ADHD
Conduct disorders
20.0
15.0
10.0
5.0
0.0
Annual excess health care cost per capita (in Euro)
Years after diagnosis
Category
Somatic services
Psychiatric services
Primary health care
Subsidised prescription

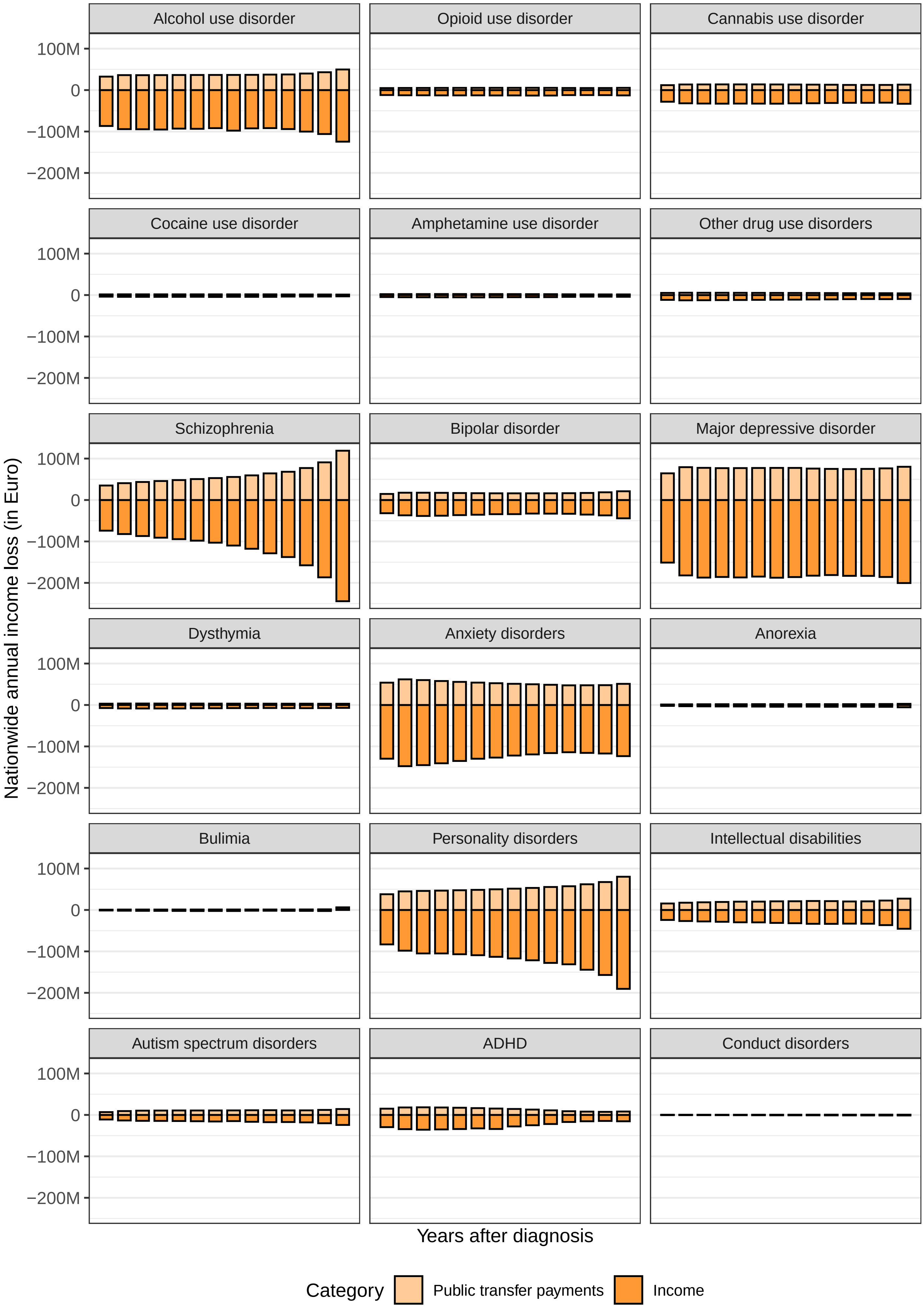

Alcohol use disorder
Opioid use disorder
Cannabis use disorder
Cocaine use disorder
Amphetamine use disorder
Other drug use disorders
Schizophrenia
Bipolar disorder
Major depressive disorder
Dysthymia
Anxiety disorders
Anorexia
Bulimia
Personality disorders
Intellectual disabilities
Autism spectrum disorders
ADHD
Conduct disorders
100M
0
−100M
−200M
Nationwide annual income loss (in Euro)
Years after diagnosis
Category
Public transfer payments
Income

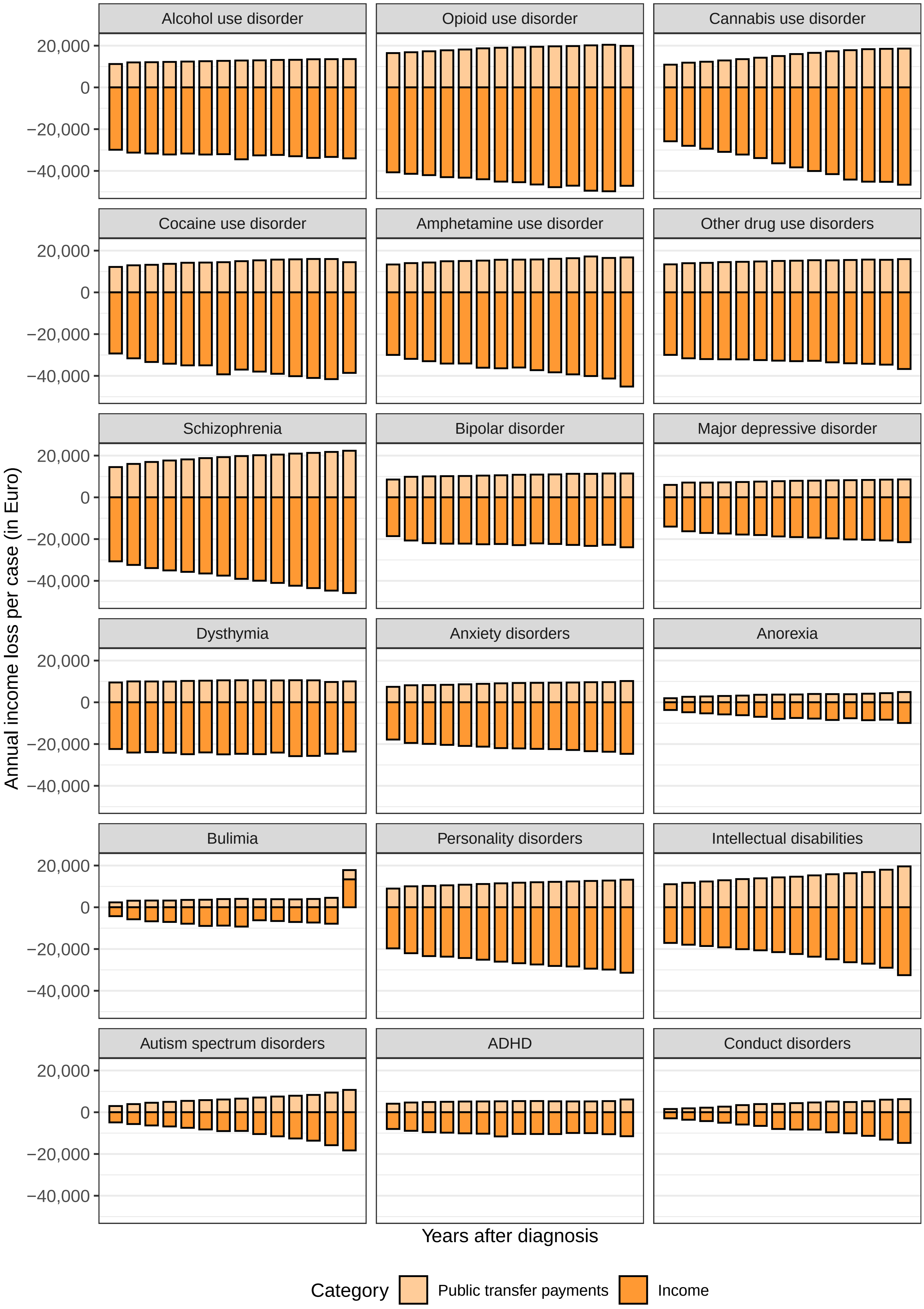

Alcohol use disorder
Opioid use disorder
Cannabis use disorder
Cocaine use disorder
Amphetamine use disorder
Other drug use disorders
Schizophrenia
Bipolar disorder
Major depressive disorder
Dysthymia
Anxiety disorders
Anorexia
Bulimia
Personality disorders
Intellectual disabilities
Autism spectrum disorders
ADHD
Conduct disorders
20,000
0
−20,000
−40,000
Annual income loss per case (in Euro)
Years after diagnosis
Category
Public transfer payments
Income

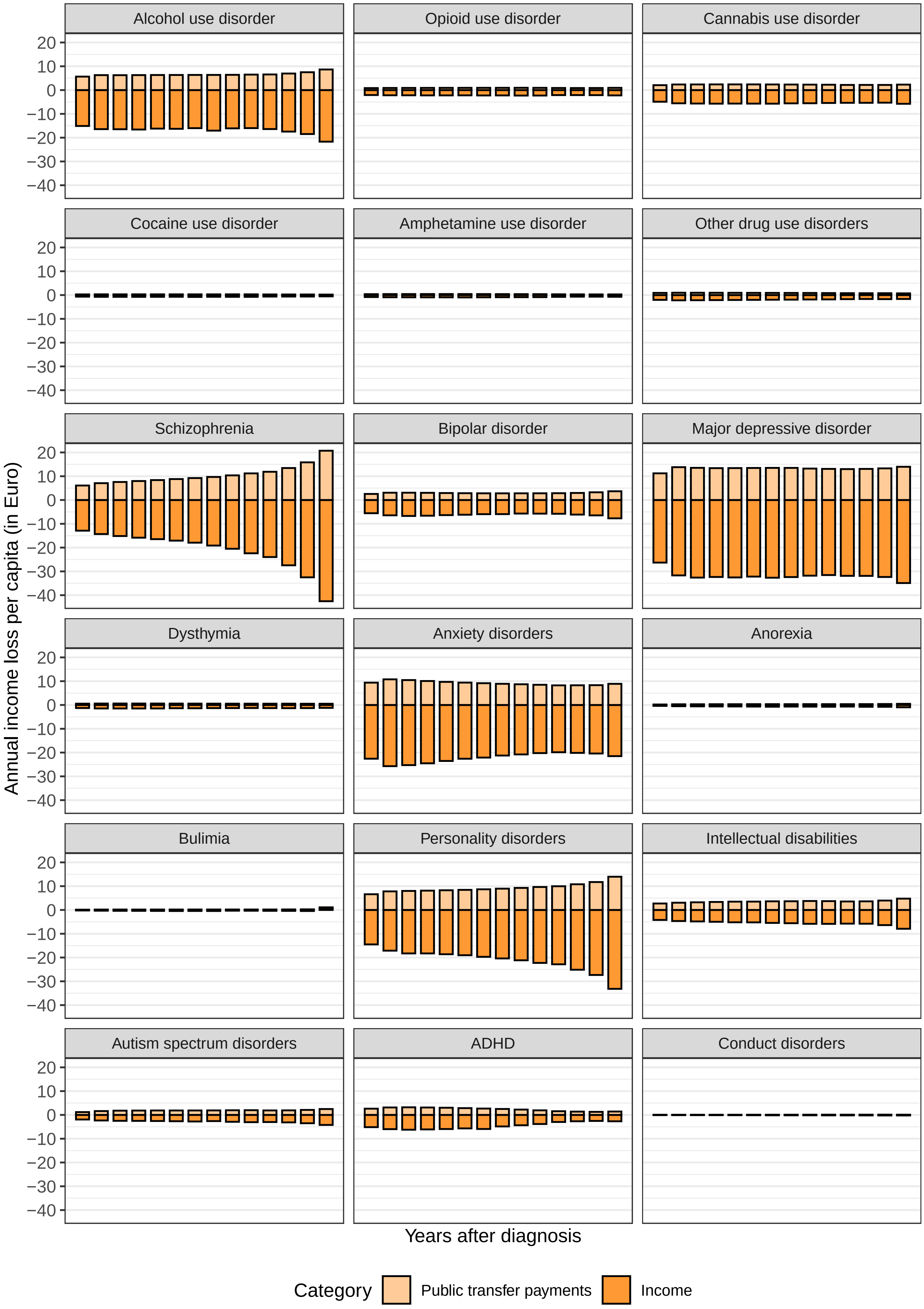
Alcohol use disorder
Opioid use disorder
Cannabis use disorder
Cocaine use disorder
Amphetamine use disorder
Other drug use disorders
Schizophrenia
Bipolar disorder
Major depressive disorder
Dysthymia
Anxiety disorders
Anorexia
Bulimia
Personality disorders
Intellectual disabilities
Autism spectrum disorders
ADHD
Conduct disorders
20
10
0
−10
−20
−30
−40
Annual income loss per capita (in Euro)
Years after diagnosis
Category
Public transfer payments
Income
